# Gene-Based Genome-Wide Association Analysis in European and Asian Populations Identified Novel Genes for Rheumatoid Arthritis

**DOI:** 10.1371/journal.pone.0167212

**Published:** 2016-11-29

**Authors:** Hong Zhu, Wei Xia, Xing-Bo Mo, Xiang Lin, Ying-Hua Qiu, Neng-Jun Yi, Yong-Hong Zhang, Fei-Yan Deng, Shu-Feng Lei

**Affiliations:** 1 Center for Genetic Epidemiology and Genomics, School of Public Health, Soochow University, Suzhou, Jiangsu, China; 2 Jiangsu Key Laboratory of Preventive and Translational Medicine for Geriatric Diseases, Soochow University, Suzhou, Jiangsu, China; 3 Department of Child and Adolescent Health, School of Public Health, Medical College of Soochow University, Suzhou, Jiangsu, China; 4 Department of Biostatistics, University of Alabama at Birmingham, Birmingham, Alabama, United States of America; University of Birmingham, UNITED KINGDOM

## Abstract

**Objective:**

Rheumatoid arthritis (RA) is a complex autoimmune disease. Using a gene-based association research strategy, the present study aims to detect unknown susceptibility to RA and to address the ethnic differences in genetic susceptibility to RA between European and Asian populations.

**Methods:**

Gene-based association analyses were performed with KGG 2.5 by using publicly available large RA datasets (14,361 RA cases and 43,923 controls of European subjects, 4,873 RA cases and 17,642 controls of Asian Subjects). For the newly identified RA-associated genes, gene set enrichment analyses and protein-protein interactions analyses were carried out with DAVID and STRING version 10.0, respectively. Differential expression verification was conducted using 4 GEO datasets. The expression levels of three selected ‘highly verified’ genes were measured by ELISA among our in-house RA cases and controls.

**Results:**

A total of 221 RA-associated genes were newly identified by gene-based association study, including 71‘overlapped’, 76 ‘European-specific’ and 74 ‘Asian-specific’ genes. Among them, 105 genes had significant differential expressions between RA patients and health controls at least in one dataset, especially for 20 genes including 11 ‘overlapped’ (*ABCF1*, *FLOT1*, *HLA-F*, *IER3*, *TUBB*, *ZKSCAN4*, *BTN3A3*, *HSP90AB1*, *CUTA*, *BRD2*, *HLA-DMA)*, 5 ‘European-specific’ *(PHTF1*, *RPS18*, *BAK1*, *TNFRSF14*, *SUOX)* and 4 ‘Asian-specific’ (*RNASET2*, *HFE*, *BTN2A2*, *MAPK13*) genes whose differential expressions were significant at least in three datasets. The protein expressions of two selected genes *FLOT1* (P value = 1.70E-02) and *HLA-DMA* (P value = 4.70E-02) in plasma were significantly different in our in-house samples.

**Conclusion:**

Our study identified 221 novel RA-associated genes and especially highlighted the importance of 20 candidate genes on RA. The results addressed ethnic genetic background differences for RA susceptibility between European and Asian populations and detected a long list of overlapped or ethnic specific RA genes. The study not only greatly increases our understanding of genetic susceptibility to RA, but also provides important insights into the ethno-genetic homogeneity and heterogeneity of RA in both ethnicities.

## Introduction

Rheumatoid arthritis (RA) is a complex autoimmune disease characterized by chronic inflammation of multiple joints, leading to progressive destruction to articular cartilage and bone. RA is strongly tied to the patients’ genetic makeup. The heritability of RA approaches 65% [1]. Extensive efforts including numerous genome-wide association studies (GWASs) so far have dramatically escalated the rate of discovery of RA-associated variants [2–4]. Recently, a genome-wide association study meta-analysis in a total of >100,000 subjects of European and Asian discovered 101 RA risk loci [5]. The SNPs identified to date, however, collectively only explain a modest proportion of the total heritability. One of possible reasons is that the traditional SNP-based GWAS used stringent thresholds of significance to control errors for the multiple testing, which resulted in a large number of SNPs with potential effects being filtered out and ignored. To help address this issue, several methods of combining P values to guide gene-level association studies were established [6–8]. Among these methods, GATES, a Simes test extension, is considerably efficient but faster and more convenient [9]. Indeed, recent studies have supported the high efficiency of gene-based association analysis in detecting disease-susceptibility genes [10–14], but currently no gene-based association study was performed to detect more novel genes for RA.

Obvious evidence has supported that substantial genetic heterogeneity exists in underlying autoimmunity among different ethnic populations. For example, the prevalence of RA is estimated to be 0.5–1.0% worldwide. However, a higher prevalence exists in populations of European ancestry than those of Asian ancestry. Among the genetic predisposition factors identified to date, *HLA-DRB1* gene is the most major determinant of RA genetic predisposition among multiple ethnic studies. But in more often situations the genes identified contributed to RA with an ethnic-specific pattern, especially for the non-HLA susceptibility genes, for example, PTPN22 gene in European populations [15,16] and PADI4 gene in Asian populations [17,18]. The detected ethnic-specific pattern may come from the inherent genetic specific differences across different ethnic populations [19,20] and also probably come from sampling biases or a lack of statistical power in the association analyses. In the era of GWASs, integrating original research results from multiethnic studies greatly improve the statistical power to uncover unknown genetic predispositions and clarify their differences in genetic background among ethnicities [21].

Therefore, based on the publicly available large RA datasets [5], this study performed high powerful gene-based association analysis to detect unknown susceptibility to RA and addressed the ethnic differences in genetic susceptibility to RA between European and Asian populations.

## Materials and Methods

### Download of the Available P Values from Previous GWASs

We first downloaded the raw P value of the genome-wide SNP-based GWAS from the publicly available Web resource http://plaza.umin.ac.jp/~yokada/datasource/software.htm[5]. The subjects in the downloaded data were enrolled from 22 GWASs (14,361 RA cases and 43,923 controls from 18 studies of Europeans, 4,873 RA cases and 17,642 controls from 4 studies of Asians). Genotyping, data-quality filter, genotype imputation of GWASs data and SNP-based association analysis were detailed in the original publication [5].

### Gene-Based Association Analysis

European-specific and Asian-specific multivariate gene-based association tests were conducted separately by using extended Simes procedure (GATES) [9].The method can use linkage disequilibrium (LD) information from a known reference population (e.g., HapMap) and therefore rapidly combine the P values of SNPs within a gene to produce valid gene-based P values without relying on raw, individual phenotype and genotype data. The standard GWAS can thus be considered a GATES preprocessing step. GATES is implemented in a systematic biological Knowledge-based mining system for Genome-wide Genetic studies (KGG 2.5) and is freely available at http://statgenpro.psychiatry.hku.hk/limx/kgg/download.php).

Steps involved in the gene-based association test were described as below: 1) Generating intermediate datasets which integrate original GWAS P values, rsID, position and chromosome column for each SNP. A total of 6,559,815 European-specific and 5,351,262 Asian-specific autosomal SNPs were used for subsequent analysis after excluding the SNPs that could not be recognized by KGG and that located in sex chromosomes (X or Y); 2) Defining a set of candidate genes of RA for the knowledge-based weighting analysis. The candidate genes here refer to genes with suggestive evidences being involved in the development of RA. We selected the 101 RA risk loci [5] corresponding genes as candidate genes. The defined length of the extended gene region is from 2-kb upstream to 2-kb downstream of each gene; 3) Conducting gene-based association test. Here, HapMap linkage disequilibrium (LD) SNP coefficients (CEU for European-specific analysis and CHB for Asian-specific analysis, downloaded from HapMap ftp:http://hapmap.ncbi.nlm.nih.gov/downloads/ld_data/2009-04_rel27/) were integrated; 4) Performing Bonferroni correction for multiple testing. According to the number of unique genes, the significant level was2.25E-06 (P = 0.05/22211) for Europeans and 2.31E-06 (P = 0.05/21609) for Asians.

To find ‘novel’ genes, we firstly excluded those genes that were also detected by the SNP-based analyses (P = 6.25E-09 for Europeans and 8.33E-09 for Asians). Then, we searched the RA-associated genes in the Phenotype-Genotype Integrator (PheGenI; http://www.ncbi.nlm.nih.gov/gap/phegeni/) by controlling P value < 1.0E-09. After excluding the genes previously identified as RA associated genes in PheGenI, the ‘novel’ genes detected by current gene-based association study were determined.

### Gene Set Enrichment Analysis and Network Pathway Analysis

To explore functional similarity of the novel RA-associated genes, we tested the probability of these genes clustering into a specific gene ontology (GO) terms and functional pathways that were defined by the Gene Ontology project and the Kyoto Encyclopedia of Genes and Genomes (KEGG) database. Specifically, the Database for Annotation, Visualization and Integrated Discovery (DAVID) integrated database query tools (http://david.d.ncifcrf.gov/) [22] was used to functionally annotate the significantly associated genes. The significance of enrichment was measured by P value according to the Fisher’s exact test and the Bonferroni correction was adopted for multiple testing. Protein-protein Interactions (PPI) among the RA-associated genes identified by gene-based association analyses were investigated by using STRING version 10.0 [23] that was freely available at http://string.embl.de.

### Differential Expression Verification of RA Associated Genes

We performed differential expression analyses for the ‘novel’ RA-associated genes identified by gene-based association study. First, we downloaded four publicly available expression datasets from GEO Datasets (www.ncbi.nlm.nih.gov/geo). These data were released in RA-related studies conducted in Caucasian subjects (GSE55235, GSE55457 and GSE15573) [24,25] and in Asian subjects (GSE17755) [26], respectively. Details on sample quality control, experiment procedures and data analyses including normalization of raw data were described in the original publications. Second, the means of the interested gene expression signals were singled out from the four datasets. Third, comparisons of mean gene expression signals between RA cases and controls were conducted separately in the four datasets through Independent-Samples T Test. P value < 0.05 was considered as significant. If the significant differential expression of one gene was verified in at least three GEO datasets, it would be determined as a ‘highly verified’ gene.

Next, the secretory genes were selected from the ‘highly verified’ ‘overlapped’ RA-associated genes for ELISA testing in our in-house sample (plasma of 25 RA patients and 13 age- and sex- matched health controls) using commercially available ELISA kits (Enzyme-linked Biotechnology Co., Ltd., Shanghai, China) according to the manufacturers’ protocols. Comparison of plasma concentrations between RA patients and controls was performed using a Mann-Whitney test. P value < 0.05 was considered significant. All patients fulfilled the American College of Rheumatology 1987/2010 revised criteria for diagnosis of RA, the average disease activity score (DAS28) of whom was 5.71. The study was approved by the Scientific Ethical Committee of the First Affiliated Hospital, Soochow University and followed the tenets of the Declaration of Helsinki. Participants in this study all provided their written informed consent.

**Fig 1:**
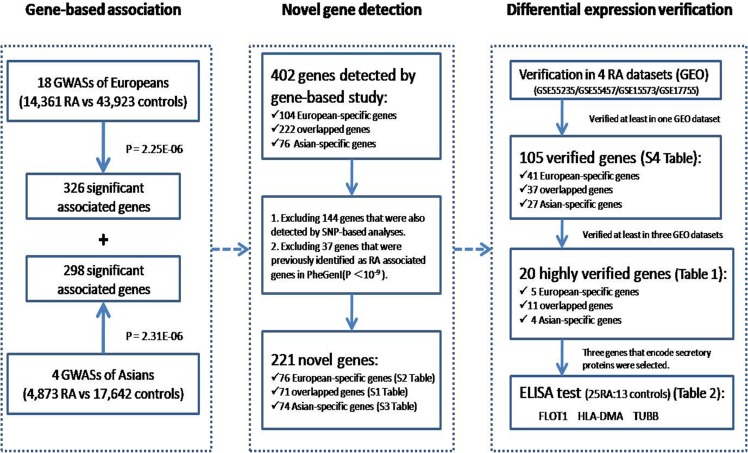
The flow chart of data analysis. European-specific and Asian-specific multivariate gene-based association tests were conducted separately by using extended Simes procedure (GATES) [9], KGG 2.5, using raw data of 18 European GWASs and 4 Asian GWASs. The 221novel genes were screened from 402gene-based detected genes. Among the 221genes, the differential expression of 105 genes was verified at least in one of four GEO datasets. The differential expressions of 20 genes were verified at least in three of four GEO datasets. Three genes encoding secretory proteins were selected from the 11 ‘highly’ verified ‘overlapped’ genes. GWASs: genome-wide association studies; PheGenI: Phenotype-Genotype Integratorhttp://www.ncbi.nlm.nih.gov/gap/phegeni/;GEO: gene expression omnibus.

The flow chart of data analysis is shown below in **Fig 1**.

## Results

### Detection of Novel Genes Associated with RA in Asians and Europeans

A total of 21,609 genes (2,562,510 SNPs inside of gene and 2,788,752 SNPs outside of gene) and 22,211 genes (3,171,781 SNPs inside of gene and 3,388,034 SNPs outside of gene) were observed in the Asian and European GWAS datasets, respectively. By comparing quantile-quantile plots (**S1 Fig**) for gene-based P value, SNP-based P value inside genes and SNP-based P value outside genes, we observed that the tail of distribution for gene-based P value was the most significant deviation both in Asian and European subjects, which suggested a relatively higher power for gene-based association analysis. The Manhattan plots of gene-level P value across chromosomes in both ethnicities were shown in **S2 Fig**.

After Bonferroni correction, 326 genes in Europeans and 298 genes in Asians were identified as RA-associated genes. Among them, 222 unique genes were overlapped in both ethnicities, 104 genes were European-specific and 76 genes were Asian-specific. To find ‘novel’ genes, we firstly excluded 144 genes that were also detected by SNP-based analyses (P = 6.25E-09 for Europeans and 8.33E-09 for Asians) (data not shown). By comparing with the RA risk genes archived in PheGenI with significant SNP-based P value < 1.0E-09, 7 ‘overlapped’ genes, 28 ‘European-specific’ genes and 2 ‘Asian-specific’ genes were excluded. Thus the remainders of 221 genes including 71 ‘overlapped’ (**S1 Table**), 76 ‘European-specific’ (**S2 Table**) and 74 ‘Asian-specific’ (**S3 Table**) genes were regarded as the newly detected genes for RA by the present study. These novel genes were not overlapped with the101 RA risk loci corresponding genes [5] that were used in defining a set of candidate genes of RA for the knowledge-based weighting analysis.

We found the ‘overlapped’ and ‘Asian-specific’ RA-associated genes were clustered within chromosome 6 (6p21, 6p22 and 6q27) while the ‘European-specific’ RA-associated genes were scattered across chromosome 1, 2, 6, 7, 9, 10,12, 17, 19, 20 and 21.Another interesting finding was that the histone 1H family genes accounted for more than one half of the ‘Asian-specific’ genes but less than one-tenth in ‘overlapped’ genes and ‘European-specific’ genes.

### Differential Expression Analyses of ‘Novel’ Detected RA Associated Genes

In the peripheral blood mononuclear cells (PBMCs) and synovial tissue of European or Asian RA patients, t-test showed that a total of 105 genes including the 37 ‘overlapped’ genes, 41 ‘European-specific’ genes and 27 ‘Asian-specific’ genes have differential expression signals (P value < 0.05) in at least one of the four functional studies (**S4 Table**). Especially, 20 genes including 11 ‘overlapped’ (*ABCF1*, *FLOT1*, *HLA-F*, *IER3*, *TUBB*, *ZKSCAN4*, *BTN3A3*, *HSP90AB1*, *CUTA*, *BRD2*, *HLA-DMA)*, 5 ‘European-specific’ *(PHTF1*, *RPS18*, *BAK1*, *TNFRSF14*, *SUOX)* and 4 ‘Asian-specific’ (*RNASET2*, *HFE*, *BTN2A2*, *MAPK13*) genes were differentially expressed between RA patients and health controls in three studies or four studies (**Table 1 and S4 Table**), and these genes were regarded as ‘highly verified’ RA-associated genes.

**Table 1:** The 20 ‘highly verified’ RA-associated genes newly identified by gene-based association study.

Gene symbol	Group	ID	Chr	Start	Stop	Map	OMIM	Description	Gene-based P value	
									European	Asian
ABCF1	Overlapped	23	6	30571392	30591531	6p21.33	603429	ATP-binding cassette, sub-family F (GCN20), member 1	1.18E-29	1.55E-14
BTN3A3*	Overlapped	10384	6	26440471	26453414	6p21.3	613595	butyrophilin, subfamily 3, member A3	5.34E-08	2.67E-10
FLOT1	Overlapped	10211	6	30742909	30727733	6p21.3	606998	flotillin 1	5.89E-20	3.87E-15
HLA-F	Overlapped	3134	6	29723339	29727295	6p21.3	143110	major histocompatibility complex, class I, F	1.03E-31	1.80E-19
IER3	Overlapped	8870	6	30744549	30743198	6p21.3	602996	immediate early response 3	3.51E-17	1.14E-15
TUBB	Overlapped	203068	6	30720379	30725421	6p21.33	191130	tubulin, beta class I	3.00E-20	4.54E-08
ZKSCAN4	Overlapped	387032	6	28259251	28244625	6p21	611643	zinc finger with KRAB and SCAN domains 4	1.71E-12	2.52E-13
BRD2*	Overlapped	6046	6	32968659	32981504	6p21.32	601540	bromodomain containing 2	1.53E-133	6.33E-07
HLA-DMA*	Overlapped	3108	6	32953121	32948613	6p21.32	142855	major histocompatibility complex, class II, DM alpha	2.75E-133	1.25E-07
HLA-G	Overlapped	3135	6	29826966	29831129	6p22.1	142871	major histocompatibility complex, class I, G	3.34E-34	1.98E-13
HSP90AB1	Overlapped	3326	6	44246165	44253887	6p21.1	140572	heat shock protein 90 alpha family class B member 1	1.87E-06	3.90E-12
PHTF1*	European-specific	10745	1	113759537	113697201	1p13	604950	putative homeodomain transcription factor 1	1.74E-147	
RPS18	European-specific	6222	6	33272074	33276503	6p21.3	180473	ribosomal protein S18	9.49E-37	
BAK1	European-specific	578	6	33580295	33572545	6p21.3	600516	BCL2-antagonist/killer 1	1.78E-10	
TNFRSF14*	European-specific	8764	1	2555766	2565621	1p36.32	602746	TNF receptor superfamily member 14	1.98E-07	
SUOX	European-specific	6821	12	55996775	56005524	12q13.2	606887	sulfite oxidase	7.10E-07	
RNASET2	Asian-specific	8635	6	167000000	167000000	6q27	612944	ribonuclease T2		6.73E-12
HFE	Asian-specific	3077	6	26087280	26096116	6p21.3	613609	hemochromatosis		2.92E-10
BTN2A2	Asian-specific	10385	6	26382869	26394873	6p22.1	613591	butyrophilin, subfamily 2, member A2		3.76E-10
MAPK13	Asian-specific	5603	6	36130483	36144523	6p21.31	602899	mitogen-activated protein kinase 13		1.40E-06

Note: Chr: chromosome; ‘highly verified’: the differential expression was verified at least in three of the four GEO datasets (GSE55235,GSE457,GSE15573 and GSE17755)

* the differential expressions of five genes (*BRD2*, *BTN3A3*, *HLA-DMA*, *PHTF1 and TNFRSF14)* were all verified in the four GEO datasets.

Further, we selected three genes *(FLOT1*, *HLA-DMA and TUBB)* that encode secretory proteins from the above 11 ‘highly verified’ ‘overlapped’ genes to test if there are differential expressions in protein level by using ELISA testing in plasma. As we expected, protein levels of FLOT1 and HLA-DMA were significantly lower in RA patients compared with health controls, but not significant for TUBB **(Table 2).**

**Table 2:** Clinical characteristics and ELISA test results of all patients and controls.

	RA patients	Controls	Mann-Whitney U	P value
**Clinical characteristics**				
Number of individuals (female: male)	25 (19:6)	13 (11:2)		
Average age (years) (range)	45.2 (21–78)	43.0 (27–67)		
DAS28 (range)	5.71 (4.87–7.20)	-		
Average ESR (mm/h)	40.76	-		
CCP antibody positive (%)	40	-		
Average CRP (mg/L) (range)	33.27 (1.5–84.7)	-		
Course of disease (year)	5.32 (0–20)	-		
**ELISA test**				
FLOT1 (pg/ml) ($\bar{X}$ ± SD)	333.72 ± 280.39	508.13 ± 301.41	85.0	0.017
HLA-DMA (ng/L) ($\bar{X}$ ± SD)	4.92 ± 5.64	7.64 ± 6.55	98.0	0.047
TUBB (ng/L) ($\bar{X}$ ± SD)	567.06 ± 396.37	713.72 ± 409.70	113.0	0.128

### Gene Set Enrichment Analysis and Network Pathway Analysis

**Fig 2:**
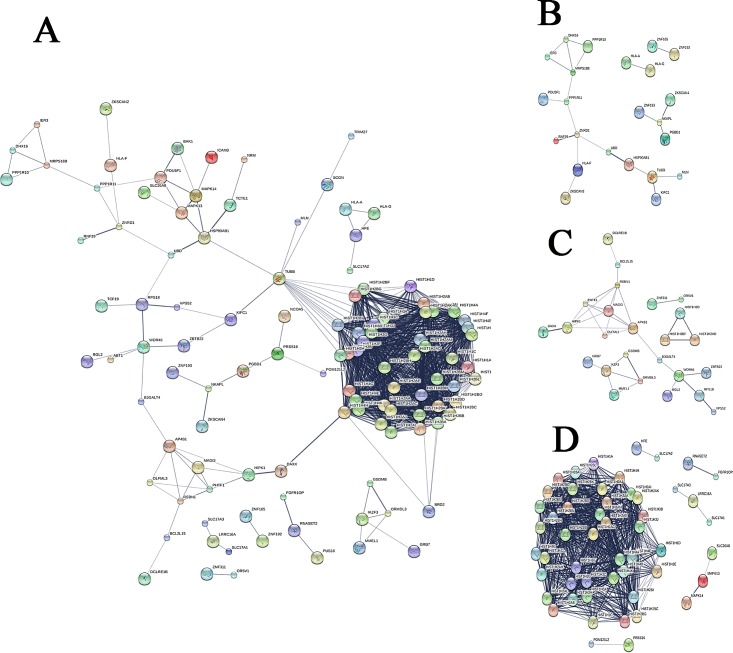
PPI network analysis for the 221‘novel’RA-associated genes. This is the confidence view of protein-protein interactions produced by STRING for(A) 221 total, (B) 71 overlapped, (C) 76 European-specific and (D)74 Asian-specific gene-based RA-associated genes whose integrated scores are bigger than 0.4. The disconnected nodes are not shown in the figure. Stronger associations are represented by thicker lines. The most visible gene set is mainly composed by histone 1H family both in (A) and (D).

For the 221 newly identified RA-associated genes, 23 GO terms and three KEGG pathways (hsa05322: Systemic lupus erythematosus, hsa05034: Alcoholism and hsa05203: Viral carcinogenesis) were significantly enriched after Bonferroni correction (**S5 and S6 Tables**). Most of the significant GO terms and pathways were related to the histone gene cluster on chromosome 6 which were enriched in ‘Asian-specific’ genes. The PPI among the newly identified RA-associated genes were showed in **Fig 2**. The most visible gene set is mainly composed by histone 1H family both in 221 total novel genes and 74 ‘Asian-specific’ genes. Most of the ‘highly verified’ RA-associated genes such as *TUBB*, *HSP90AB1*, *RPS18*, *BRD2*, *PHTF1*, *MAPK13*, *BAK1*, *HLA-F*, *IER3*, *RNASET2*, *HLA-G*, *ZKSCAN4* and *HFE* were showed in the STRING Network Visualization.

## Discussion

In this study we performed the gene-based GWASs association tests using the publicly available datasets of the largest combining GWASs. The gene-based analysis has the following advantages: 1) genes, not SNPs, are thought to be the functional units in the genome; 2) genes rather than SNPs are highly consistent across diverse populations; 3) gene-based analyses rather than SNP-based analyses in GWASs can alleviate the multiple testing burden and thus improves the statistical power to detect significant genes; 4) candidate genes identified by gene-based association study are directly suitable for further pathway and network-based analysis. When doing SNP-based study, KGG prioritizes SNPs through a knowledge-based weighting method which can maximize the potential power of association tests while controlling false positive discoveries rate and thus could detect more candidate genes. The gene-based association study identified 402 RA-susceptibility genes even after very strict Bonferroni corrections. More importantly, after excluding the known RA-associated genes, the present study discovered 221 ‘novel’ RA genes. Near half of the 221 novel genes (105 genes) had significant differential expression signals between RA patients and health controls in the next functional validation tests, among which twenty genes were highly verified. All these evidences highlighted the relatively higher power for the gene-based association analysis.

An important topic of this study is the ethnogenetic homogeneity and heterogeneity in RA etiology. We provide evidence of 71 ‘overlapped’ RA risk genes in Asian and European individuals. Among them, 37 genes have differential expression signals (P value < 0.05) in synovial tissues or PBMCs of RA patients of Asian and European, and,11 genes (*ABCF1*, *FLOT1*, *HLA-F*, *IER3*, *TUBB*, *ZKSCAN4*,*BTN3A3*, *HSP90AB1*, *BRD2*, *HLA-G and HLA-DMA*) are highly verified in three or four functional studies. These observations support the view that the genetic risk of RA is shared, in general, among Asians and Europeans [5,27]. We also highlight apparent differences across ethnic groups. First, there are 74 ‘Asian-specific’ and 76 ‘European-specific’ RA risk genes detected by our gene-based association study, suggesting that ethnic variation should be considered in RA etiology. Second, the ‘Asian-specific’ RA risk genes are clustered together within chromosome 6 while the ‘European-specific’ RA risk genes are scattered across multiple chromosomes, which means that multiple risk genes scatted in the genome may contribute RA pathogenesis even if they are not the primary causes, and, Europeans may have more diverse genetic heterogeneity in RA etiology. Third, more than half of the newly identified ‘Asian-specific’ genes are histone 1H family genes, which accounts for less than one-tenth in ‘European-specific’ genes. It is commonly known that histones play a central role in transcription regulation, DNA repair, DNA replication and chromosomal stability. However, there are few reports about the relationship of histone family and RA. It is a novel finding that the histone 1H family is associated with RA in Asian population. Fourth, although a total of 27 ‘Asian-specific’ and 41 ‘European-specific’ newly identified genes are differentially expressed between RA patients and controls, only two ‘European-specific’ genes, *PHTF1* and *TNFRSF14*, are validated by all the four functional studies, and, only PHTF1 shows an opposite RA/control ratios of mean expression value between Europeans and Asians. This hints that we might need to consider both the tissue specificity and race specificity when making functional verification tests.

Another interesting finding is that most of the ‘highly verified’ RA-associated genes might have potential connections with RA pathogenesis. For instance, *BRD2*, the ‘European-specific’ RA-associated gene, is directly connected with the histone 1H cluster in the confidence view of STRING. Although the functional relationship of *BRD2* and RA is unclear till now, it is reported that Bromodomains (BRDs) are protein interaction modules that exclusively recognize acetylation motifs [28] and there is a structural basis for deciphering the histone code by BRD2 through the binding with a long segment of the histone H4 tail and then presumably prevent erasure of the histone code during the cell cycle [29]. As for *HLA-DMA*, it is another highly verified RA risk gene both in European and Asian populations. It plays a critical role in catalyzing the release of class II HLA-associated invariant chain-derived peptides from newly synthesized class II HLA molecules and freeing the peptide binding site for acquisition of antigenic peptides [30]. Given that a striking association is found between RA and particular *HLA-DRB1*, it seems to be a good candidate allele involved in RA pathogenesis [31]. However, it is previously reported that the *HLA-DM* (DMA and DMB) genes do not have any influences on their own to genetic susceptibility to RA [32,33]. More in-depth work is necessary to determine whether *HLA-DMA* is indeed associated with RA. With regard to *PHTF1*and *BTN3A3*, the highly verified ‘European-specific’ and ‘overlapped’ RA risk gene, no direct evidence has been reported till now that they are involved in RA etiology. *PHTF1* (putative homeodomain transcriptional factor), a putative homeobox gene located at band 1p11-p13 of the human genome, may play a role in transcription regulation. It encodes a membrane protein abundantly expressed in male germinal cells [34]. The rs6679677 (*PHTF1-PTPN22*) is reported as a susceptibility factor for autoimmunity in diabetes type 1 [35,36] while *PTPN22* is a well-known RA risk gene. *BTN3A3* (Butyrophilin, Subfamily 3, Member A3), also called *CD277*, belongs to the B7 family members and is expressed in various immune cells such as T and NK cells [37]. *BTN3A3*may act as one of the inhibitors of co-stimulation for T lymphocyte priming, similar to *CTLA-4* [38]. It also found that SNPs near the butyrophilin genes (*BTN3A3/BTN2A1*) are associated with variations in IFN-γ secretion [39]. As for *FLOT1* (flotillin-1), another gene verified by our ELISA test, its important roles in promoting tumorigenesis and progression of several cancers like non-small cell lung cancer, breast cancer and hepatocellular carcinoma have been recently reported [40,41]. The function of flotillin 1 in RA development has not been determined. However it is found that FLOT1 can activate tumor necrosis factor-alpha (TNF-α) receptor signaling and sustain activation of NF-kappa B in esophageal squamous cell carcinoma cells [42]. Taken together, the above evidence mentioned supports that the ‘highly verified’ RA-associated genes are worth in-depth study. Further studies are needed on a number of issues including how histone 1H genes relate to RA, whether the newly identified candidate genes especially those highly verified genes truly relate to RA etiology, and, if any, what functional relationships are between these genes and RA.

Our study has several limitations. The gene-based association analyses of combining GWASs did not include the SNPs in X/Y chromosomes or that could not be recognized by KGG, thus the significant genes might not be fully detected. The sample size of our functional differential expression analyses was relatively small. Since only plasma for the subjects is available for us and our budget is limited, we could only select three secretory genes from the eleven ‘highly verified ‘‘overlapped’ RA-associated genes for ELISA test and left a long list of candidate genes to be tested in protein level.

In conclusion, using the gene-based association research strategy, our study identified a long list of novel RA associated genes and also addressed their ethno-genetic homogeneity and heterogeneity in European and Asian populations. Our findings point to the involvement of novel genes and pathways in the pathogenesis of RA, and provide more insights into ethnic differences in genetic susceptibility to RA between European and Asian populations.

## Supporting Information

S1 Fig **Quantile-quantile plots in a) Asians and b) Europeans.** There are three Quantile-quantile plots of the observed P value distributions in each diagram, namely the gene-based P value, the original SNP inside of gene P value and the SNPs outside of gene P value. The x-axis indicates the expected–log_10_ (P values). The y-axis indicates the observed—log_10_ (P values) after the application of gene association analysis. From left to right in order, the association results of gene P value, SNPs inside of gene P value and SNPs outside of gene P value are indicated, respectively. As compared with the expected null P value distributions, the tail of the distribution for gene-based P value is the most significant deviation both in populations of **a)** Asians and **b)** Europeans.
(DOCX)

S2 Fig **Manhattan plots of gene P values (chromosome 1 to 22) in Asians (a and b) and Europeans (c and d).** The y-axis indicates the–log10 (P value) of genome-wide genes in each GWAS association analysis. In order to present the whole genome clearly, two plots were drawn for chromosome 1 to 8, and chromosome 9 to 22, respectively. The genes for which P values were less than 1.0E-10 are not indicated.
(DOCX)

S1 Table The 71 RA-associated genes ‘overlapped’ in Asians and Europeans and newly detected by gene-based association study. Note: ‘Chr’: Chromosome, ‘-‘: not available, ‘Start’ and ‘stop’: Genomic Location.
(DOCX)

S2 Table The 76 ‘European-specific’ RA-associated genes newly detected by gene-based association study. Note: ‘Chr’: Chromosome, ‘-‘: not available, ‘Start’ and ‘stop’: Genomic Location.
(DOCX)

S3 Table The 74 ‘Asian-specific’ RA-associated genes newly detected by gene-based association study. Note: ‘Chr’: Chromosome, ‘-‘: not available, ‘Start’ and ‘stop’: Genomic Location
(DOCX)

S4 Table Differential expression analyses for the ‘novel’ RA-associated genes identified by gene-based study. Note: RA: rheumatoid arthritis; HC: health controls; PBMC: peripheral blood mononuclear cell; GSE number: Gene Expression Omnibus, http://www.ncbi.nlm.nih.gov/geo/; ★t overlapped genes; ◆o European-specific genes; ●u Asian-specific genes. We only listed the most significant expression results of probes if one gene has multiple detected probes.
(DOCX)

S5 Table Functional annotation clustering analysis for the 221 newly Identified RA-associated Genes. Note: Functional annotation clustering analysis was performed using DAVID.
(DOCX)

S6 Table Functional annotation clustering analysis for the ‘overlapped’, ‘European-specific’, and ‘Asian-specific’ RA-associated genes. Note: Functional annotation clustering analysis was performed using STRING.
(DOCX)
