# Supplementary figures and images for: Gene-Based Genome-Wide Association Analysis in European and Asian Populations Identified Novel Genes for Rheumatoid Arthritis
Source: PLoS One. 2016 Nov 29;11(11):e0167212. doi: 10.1371/journal.pone.0167212 (PMC5127563; doi:10.1371/journal.pone.0167212)

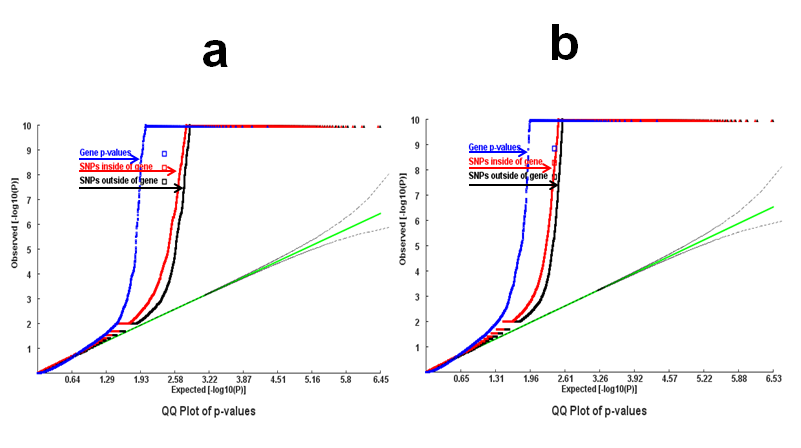


**Figure S1. Quantile-quantile plots in a) Asians and b) Europeans.**

Supplement: S1 Fig — Quantile-quantile plots in a) Asians and b) Europeans. There are three Quantile-quantile plots of the observed P value distributions in each diagram, namely the gene-based P value, the original SNP inside of gene P value and the SNPs outside of gene P value. The x-axis indicates the expected–log10 (P values). The y-axis indicates the observed—log10 (P values) after the application of gene association analysis. From left to right in order, the association results of gene P value, SNPs inside of gene P value and SNPs outside of gene P value are indicated, respectively. As compared with the expected null P value distributions, the tail of the distribution for gene-based P value is the most significant deviation both in populations of a) Asians and b) Europeans. (DOCX) [file pone.0167212.s001.docx]

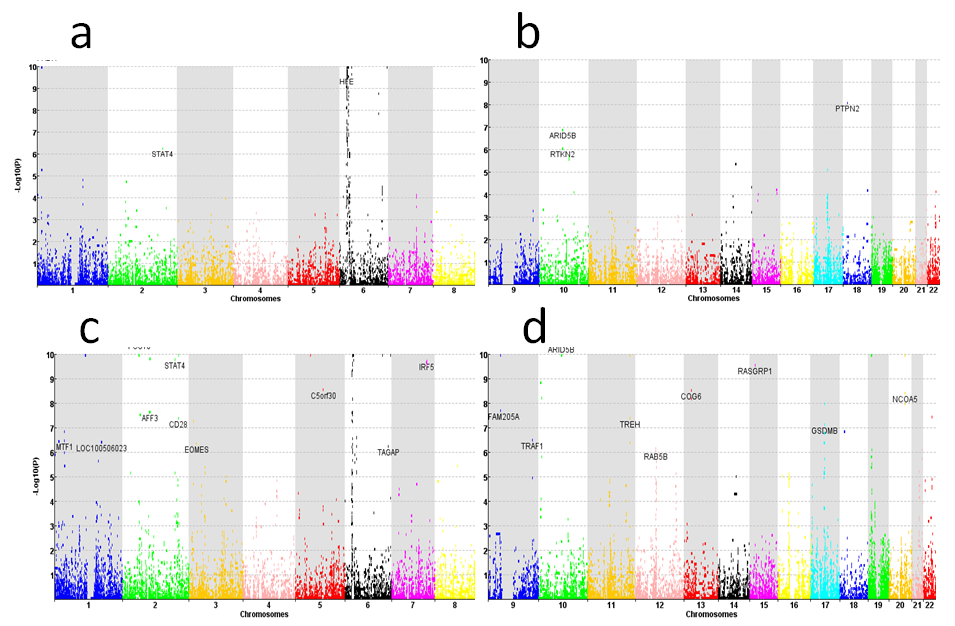


**Figure S2. Manhattan plots of gene P values (chromosome 1 to 22) in Asians (a and b) and Europeans (c and d).**

Supplement: S2 Fig — Manhattan plots of gene P values (chromosome 1 to 22) in Asians (a and b) and Europeans (c and d). The y-axis indicates the–log10 (P value) of genome-wide genes in each GWAS association analysis. In order to present the whole genome clearly, two plots were drawn for chromosome 1 to 8, and chromosome 9 to 22, respectively. The genes for which P values were less than 1.0E-10 are not indicated. (DOCX) [file pone.0167212.s002.docx]
