# Supplementary material for: Gene-Based Genome-Wide Association Analysis in European and Asian Populations Identified Novel Genes for Rheumatoid Arthritis
Source: PLoS One. 2016 Nov 29;11(11):e0167212. doi: 10.1371/journal.pone.0167212 (PMC5127563; doi:10.1371/journal.pone.0167212)
Supplement: S1 Table — Note: ‘Chr’: Chromosome, ‘-‘: not available, ‘Start’ and ‘stop’: Genomic Location. (DOCX) [file pone.0167212.s003.docx]

**Table S1. The 71 RA-associated genes ‘overlapped’ in Asians and Europeans and newly detected by gene-based association study**

| **Gene symbol** | **ID** | **Chr** | **Start** | **Stop** | **Map** | **OMIM** | **Description** | **P value** | |
| --- | --- | --- | --- | --- | --- | --- | --- | --- | --- |
|  |  |  |  |  |  |  |  | **European** | **Asian** |
| ABCF1 | 23 | 6 | 30571392 | 30591531 | 6p21.33 | 603429 | ATP-binding cassette, sub-family F (GCN20), member 1 | 1.18E-29 | 1.55E-14 |
| ATAT1 | 79969 | 6 | 30626835 | 30646820 | 6p21.33 | 615556 | alpha tubulin acetyltransferase 1 | 3.88E-30 | 2.26E-11 |
| BTN3A1 | 11119 | 6 | 26402236 | 26415215 | 6p22.1 | 613593 | butyrophilin, subfamily 3, member A1 | 2.48E-08 | 2.38E-10 |
| BTN3A3 | 10384 | 6 | 26440471 | 26453414 | 6p21.3 | 613595 | butyrophilin, subfamily 3, member A3 | 5.34E-08 | 2.67E-10 |
| C6orf100 | 729583 | 6 | 28943783 | 28944537 | 6p22.1 | - | long intergenic non-protein coding RNA 1556 | 4.46E-16 | 3.87E-12 |
| C6orf136 | 221545 | 6 | 30647038 | 30653209 | 6p21.33 | - | chromosome 6 open reading frame 136 | 1.79E-30 | 5.41E-12 |
| CDSN | 1041 | 6 | 31120474 | 31115087 | 6p21.3 | 602593 | corneodesmosin | 3.37E-32 | 1.52E-22 |
| DHX16 | 8449 | 6 | 30673052 | 30653118 | 6p21.3 | 603405 | DEAH (Asp-Glu-Ala-His) box polypeptide 16 | 3.78E-20 | 5.87E-11 |
| FLOT1 | 10211 | 6 | 30742909 | 30727733 | 6p21.3 | 606998 | flotillin 1 | 5.89E-20 | 3.87E-15 |
| GPX5 | 2880 | 6 | 28526011 | 28534950 | 6p22.1 | 603435 | glutathione peroxidase 5 | 3.16E-13 | 7.20E-13 |
| HCG4 | 54435 | 6 | 29793072 | 29791030 | 6p21.3 | - | HLA complex group 4 (non-protein coding) | 3.34E-32 | 3.33E-17 |
| HCG4B | 80868 | 6 | 29927214 | 29924591 | 6p21.3 | - | HLA complex group 4B (non-protein coding) | 3.09E-26 | 7.06E-15 |
| HCG8 | 100507399 | 6 | 30013921 | 30012100 | 6p21.3 | 615801 | HLA complex group 8 | 2.10E-32 | 1.19E-12 |
| HCG9 | 10255 | 6 | 29975114 | 29978402 | 6p21.3 | 615797 | HLA complex group 9 (non-protein coding) | 5.18E-33 | 1.35E-13 |
| HLA-A | 3105 | 6 | 29942469 | 29945883 | 6p21.3 | 142800 | major histocompatibility complex, class I, A | 4.96E-33 | 9.45E-14 |
| HLA-F | 3134 | 6 | 29723339 | 29727295 | 6p21.3 | 143110 | major histocompatibility complex, class I, F | 1.03E-31 | 1.80E-19 |
| HLA-J | 3137 | 6 | 30005970 | 30009955 | 6p21.31 | - | major histocompatibility complex, class I, J (pseudogene) | 8.14E-32 | 2.29E-12 |
| IER3 | 8870 | 6 | 30744549 | 30743198 | 6p21.3 | 602996 | immediate early response 3 | 3.51E-17 | 1.14E-15 |
| KIFC1 | 3833 | 6 | 33391535 | 33409923 | 6p21.3 | 603763 | kinesin family member C1 | 5.03E-24 | 1.04E-08 |
| LEMD2 | 221496 | 6 | 33789128 | 33771212 | 6p21.31 | 616312 | LEM domain containing 2 | 7.94E-30 | 5.73E-19 |
| LINC01015 | 100507362 | 6 | 29529405 | 29533567 | 6p21.33 | - | long intergenic non-protein coding RNA 1015 | 6.63E-09 | 1.19E-22 |
| LOC100130476 | 100130476 | 6 | 137868232 | 137823668 | 6q23.3 | - | uncharacterized LOC100130476 | 6.84E-15 | 1.48E-08 |
| LOC554223 | 554223 | 6 | 29791905 | 29797806 | 6p21.3 | - | histocompatibility antigen-related | 3.34E-32 | 3.33E-17 |
| MAS1L | 116511 | 6 | 29487901 | 29486765 | 6p22.1 | 607235 | MAS1 proto-oncogene like, G protein-coupled receptor | 7.22E-14 | 1.24E-20 |
| MIR877 | 100126314 | 6 | 30584331 | 30584416 | 6p21.33 | 611619 | microRNA 877 | 1.89E-16 | 3.50E-09 |
| MLN | 4295 | 6 | 33804015 | 33794671 | 6p21.3 | 158270 | motilin | 2.62E-30 | 9.80E-13 |
| MRPS18B | 28973 | 6 | 30617497 | 30626396 | 6p21.3 | 611982 | mitochondrial ribosomal protein S18B | 1.46E-19 | 2.78E-10 |
| NKAPL | 222698 | 6 | 28259296 | 28260957 | 6p22.1 | - | NFKB activating protein-like | 3.55E-12 | 2.37E-13 |
| NRM | 11270 | 6 | 30691419 | 30688046 | 6p21.33 | - | nurim (nuclear envelope membrane protein) | 2.39E-22 | 1.98E-13 |
| OR10C1 | 442194 | 6 | 29439938 | 29440976 | 6p22.1 | - | olfactory receptor, family 10, subfamily C, member 1 (gene/pseudogene) | 7.19E-09 | 1.20E-19 |
| OR11A1 | 26531 | 6 | 29457070 | 29425503 | 6p22.2-p21.31 | - | olfactory receptor, family 11, subfamily A, member 1 | 2.40E-08 | 1.64E-15 |
| OR12D2 | 26529 | 6 | 29396638 | 29397670 | 6p22.2-p21.31 | - | olfactory receptor, family 12, subfamily D, member 2 (gene/pseudogene) | 1.25E-13 | 4.06E-15 |
| OR2B6 | 26212 | 6 | 27957240 | 27958181 | 6p21.3 | - | olfactory receptor, family 2, subfamily B, member 6 | 3.13E-08 | 2.75E-11 |
| PGBD1 | 84547 | 6 | 28281536 | 28302548 | 6p22.1 | - | piggyBac transposable element derived 1 | 9.94E-14 | 4.42E-13 |
| POU5F1 | 5460 | 6 | 31170692 | 31164336 | 6p21.31 | 164177 | POU class 5 homeobox 1 | 4.69E-33 | 8.76E-15 |
| PPP1R10 | 5514 | 6 | 30618606 | 30600399 | 6p21.3 | 603771 | protein phosphatase 1, regulatory subunit 10 | 1.72E-32 | 2.77E-14 |
| PPP1R11 | 6992 | 6 | 30066693 | 30070332 | 6p21.3 | 606670 | protein phosphatase 1, regulatory (inhibitor) subunit 11 | 5.41E-34 | 6.15E-20 |
| PPP1R18 | 170954 | 6 | 30687894 | 30676388 | 6p21.3 | 610990 | protein phosphatase 1, regulatory subunit 18 | 6.40E-22 | 3.61E-13 |
| PRR3 | 80742 | 6 | 30556708 | 30564695 | 6p21.33 | - | proline rich 3 | 2.88E-30 | 7.49E-15 |
| PSORS1C3 | 100130889 | 6 | 31177898 | 31173734 | 6p21.33 | - | psoriasis susceptibility 1 candidate 3 (non-protein coding) | 3.86E-33 | 8.14E-14 |
| RNF39 | 80352 | 6 | 30075850 | 30070265 | 6p21.3 | 607524 | ring finger protein 39 | 3.49E-34 | 1.07E-19 |
| TCF19 | 6941 | 6 | 31158525 | 31167160 | 6p21.3 | 600912 | transcription factor 19 | 4.35E-33 | 6.85E-15 |
| TOB2P1 | 222699 | 6 | 28218928 | 28215337 | 6p22.1 | - | transducer of ERBB2, 2 pseudogene 1 | 5.89E-08 | 2.26E-12 |
| TUBB | 203068 | 6 | 30720379 | 30725421 | 6p21.33 | 191130 | tubulin, beta class I | 3.00E-20 | 4.54E-08 |
| UBD | 10537 | 6 | 29559924 | 29555611 | 6p21.3 | 606050 | ubiquitin D | 3.66E-21 | 2.71E-23 |
| UQCC2 | 84300 | 6 | 33711750 | 33696760 | 6p21.31 | 614461 | ubiquinol-cytochrome c reductase complex assembly factor 2 | 1.50E-30 | 8.68E-12 |
| ZKSCAN3 | 80317 | 6 | 28349912 | 28369176 | 6p22.1 | 612791 | zinc finger with KRAB and SCAN domains 3 | 4.75E-17 | 7.53E-13 |
| ZKSCAN4 | 387032 | 6 | 28259251 | 28244625 | 6p21 | 611643 | zinc finger with KRAB and SCAN domains 4 | 1.71E-12 | 2.52E-13 |
| ZNF165 | 7718 | 6 | 28080703 | 28089562 | 6p21.3 | 600834 | zinc finger protein 165 | 3.96E-11 | 2.56E-10 |
| ZNF192P1 | 651302 | 6 | 28161760 | 28169594 | 6p22.1 | - | zinc finger protein 192 pseudogene 1 | 1.16E-10 | 3.39E-10 |
| ZNRD1 | 30834 | 6 | 30061239 | 30064908 | 6p21.3 | 607525 | zinc ribbon domain containing 1 | 4.30E-34 | 5.39E-20 |
| ZNRD1-AS1 | 80862 | 6 | 30061183 | 30001010 | 6p22.1 | 615714 | ZNRD1 antisense RNA 1 | 1.86E-33 | 1.49E-19 |
| ZSCAN12 | 9753 | 6 | 28399766 | 28378820 | 6p21 | 603978 | zinc finger and SCAN domain containing 12 | 3.99E-17 | 1.06E-12 |
| ZSCAN12P1 | 221584 | 6 | 28090806 | 28095714 | 6p22.1 | - | zinc finger and SCAN domain containing 12 pseudogene 1 | 7.98E-12 | 1.93E-10 |
| ZSCAN26 | 7741 | 6 | 28267009 | 28278223 | 6p21.31 | - | zinc finger and SCAN domain containing 26 | 5.01E-14 | 4.98E-13 |
| ZSCAN31 | 64288 | 6 | 28337365 | 28324736 | 6p22.3-p22.1 | 610794 | zinc finger and SCAN domain containing 31 | 5.05E-11 | 2.91E-13 |
| ZSCAN9 | 7746 | 6 | 28225250 | 28233486 | 6p21.3 | 602246 | zinc finger and SCAN domain containing 9 | 4.30E-13 | 6.78E-13 |
| BRD2 | 6046 | 6 | 32968659 | 32981504 | 6p21.32 | 601540 | bromodomain containing 2 | 1.53E-133 | 6.33E-07 |
| HIST1H4E | 8367 | 6 | 26204644 | 26205020 | 6p22.2 | 602830 | histone cluster 1 H4 family member e | 7.94E-10 | 1.75E-07 |
| HLA-DMA | 3108 | 6 | 32953121 | 32948613 | 6p21.32 | 142855 | major histocompatibility complex, class II, DM alpha | 2.75E-133 | 1.25E-07 |
| HLA-G | 3135 | 6 | 29826966 | 29831129 | 6p22.1 | 142871 | major histocompatibility complex, class I, G | 3.34E-34 | 1.98E-13 |
| HSP90AB1 | 3326 | 6 | 44246165 | 44253887 | 6p21.1 | 140572 | heat shock protein 90 alpha family class B member 1 | 1.87E-06 | 3.90E-12 |
| LINC01016 | 100507584 | 6 | 33896906 | 33889510 | 6p21.31 | - | long intergenic non-protein coding RNA 1016 | 4.66E-13 | 1.44E-07 |
| MIR4647 | 100616124 | 6 | 44254284 | 44254205 | 6p21.1 | - | microRNA 4647 | 5.89E-07 | 1.08E-11 |
| SLC35B2 | 347734 | 6 | 44257889 | 44254100 | 6p21.1 | 610788 | solute carrier family 35 member B2 | 4.81E-07 | 8.10E-13 |
| SYNGAP1 | 8831 | 6 | 33420069 | 33453688 | 6p21.32 | 603384 | synaptic Ras GTPase activating protein 1 | 2.69E-07 | 4.33E-12 |
| TMEM151B | 441151 | 6 | 44270742 | 44279444 | 6p21.1 | - | transmembrane protein 151B | 9.20E-91 | 6.69E-07 |
| ZBED9 | 114821 | 6 | 28616202 | 28568287 | 6p22.1 | 615254 | zinc finger BED-type containing 9 | 2.57E-07 | 1.93E-12 |
| ZKSCAN8 | 7745 | 6 | 28141643 | 28159471 | 6p22.1 | 602240 | zinc finger with KRAB and SCAN domains 8 | 1.24E-10 | 6.26E-07 |
| ZSCAN16 | 80345 | 6 | 28123752 | 28130085 | 6p22.1 | - | zinc finger and SCAN domain containing 16 | 8.24E-07 | 1.03E-10 |
| ZSCAN16-AS1 | 100129195 | 6 | 28137315 | 28121794 | 6p22.1 | - | ZSCAN16 antisense RNA 1 | 1.10E-06 | 1.55E-10 |

Note: ‘Chr’: Chromosome, ‘-‘: not available, ‘Start’ and ‘stop’: Genomic Location
