# Supplementary material for: Gene-Based Genome-Wide Association Analysis in European and Asian Populations Identified Novel Genes for Rheumatoid Arthritis
Source: PLoS One. 2016 Nov 29;11(11):e0167212. doi: 10.1371/journal.pone.0167212 (PMC5127563; doi:10.1371/journal.pone.0167212)
Supplement: S2 Table — Note: ‘Chr’: Chromosome, ‘-‘: not available, ‘Start’ and ‘stop’: Genomic Location. (DOCX) [file pone.0167212.s004.docx]

**Table S2. The 76 ‘European-specific’ RA-associated genes newly detected by gene-based association study**

| **Gene symbol** | **ID** | **Chr** | **Start** | **Stop** | **Map** | **OMIM** | **Description** | **P value** |
| --- | --- | --- | --- | --- | --- | --- | --- | --- |
| RSBN1 | 54665 | 1 | 113812475 | 113761831 | 1p13.2 | 615858 | round spermatid basic protein 1 | 1.18E-147 |
| PHTF1 | 10745 | 1 | 113759537 | 113697201 | 1p13 | 604950 | putative homeodomain transcription factor 1 | 1.74E-147 |
| MAGI3 | 260425 | 1 | 113390464 | 113685922 | 1p12-p11.2 | 615943 | membrane associated guanylate kinase, WW and PDZ domain containing 3 | 2.18E-93 |
| ZBTB9 | 221504 | 6 | 33454578 | 33457543 | 6p21.32 | - | zinc finger and BTB domain containing 9 | 1.68E-91 |
| BCL2L15 | 440603 | 1 | 113887607 | 113876813 | 1p13.2 | - | BCL2-like 15 | 5.67E-65 |
| AP4B1-AS1 | 100287722 | 1 | 113814386 | 113869961 | 1p13.2 | - | AP4B1 antisense RNA 1 | 2.02E-64 |
| AP4B1 | 10717 | 1 | 113905118 | 113894747 | 1p13.2 | 607245 | adaptor-related protein complex 4, beta 1 subunit | 1.19E-61 |
| DCLRE1B | 64858 | 1 | 113905292 | 113914085 | 1p13.2 | 609683 | DNA cross-link repair 1B | 1.69E-61 |
| CUTA | 51596 | 6 | 33418287 | 33416541 | 6p21.32 | 616953 | cutA divalent cation tolerance homolog (E. coli) | 9.3E-58 |
| HIPK1 | 204851 | 1 | 113929196 | 113977868 | 1p13.2 | 608003 | homeodomain interacting protein kinase 1 | 9.65E-50 |
| DAXX | 1616 | 6 | 33323015 | 33318557 | 6p21.3 | 603186 | death-domain associated protein | 4.41E-43 |
| HCG25 | 414765 | 6 | 33249535 | 33254889 | 6p21 | - | HLA complex group 25 (non-protein coding) | 1.72E-42 |
| RGL2 | 5863 | 6 | 33299459 | 33291653 | 6p21.3 | 602306 | ral guanine nucleotide dissociation stimulator-like 2 | 2.63E-42 |
| VPS52 | 6293 | 6 | 33271964 | 33250271 | 6p21.3 | 603443 | vacuolar protein sorting 52 homolog (S. cerevisiae) | 3.59E-42 |
| PFDN6 | 10471 | 6 | 33289596 | 33290933 | 6p21.3 | 605660 | prefoldin subunit 6 | 9.05E-41 |
| WDR46 | 9277 | 6 | 33289526 | 33278904 | 6p21.3 | 611440 | WD repeat domain 46 | 1.32E-40 |
| GGNBP1 | 449520 | 6 | 33583698 | 33589025 | 6p21 | 609495 | gametogenetin binding protein 1 (pseudogene) | 7.14E-37 |
| LINC00336 | 401253 | 6 | 33593337 | 33586105 | 6p21.31 | - | long intergenic non-protein coding RNA 336 | 7.14E-37 |
| RPS18 | 6222 | 6 | 33272074 | 33276503 | 6p21.3 | 180473 | ribosomal protein S18 | 9.49E-37 |
| OLFML3 | 56944 | 1 | 113979390 | 113982253 | 1p13.2 | 610088 | olfactomedin-like 3 | 2.01E-36 |
| B3GALT4 | 8705 | 6 | 33277139 | 33278824 | 6p21.3 | 603095 | UDP-Gal:betaGlcNAc beta 1,3-galactosyltransferase, polypeptide 4 | 9.63E-36 |
| MIR5004 | 100847012 | 6 | 33438330 | 33438436 |  | - | microRNA 5004 | 2.9E-30 |
| ZBTB22 | 9278 | 6 | 33317941 | 33314404 | 6p21.3 | 611439 | zinc finger and BTB domain containing 22 | 4.01E-27 |
| HLA-H | 3136 | 6 | 29887759 | 29891079 | 6p21.3 | 142800 | major histocompatibility complex, class I, H (pseudogene) | 2.51E-19 |
| HCG14 | 414760 | 6 | 28896529 | 28897319 | 6p21 | - | HLA complex group 14 (non-protein coding) | 3.24E-17 |
| OR2H2 | 7932 | 6 | 29586040 | 29588967 | 6p21.3 | 600578 | olfactory receptor, family 2, subfamily H, member 2 | 1.8E-15 |
| ZNF311 | 282890 | 6 | 29005605 | 28994784 | 6p22.1 | - | zinc finger protein 311 | 2.24E-15 |
| OR14J1 | 442191 | 6 | 29306689 | 29307654 | 6p22.1 | - | olfactory receptor, family 14, subfamily J, member 1 | 2.27E-14 |
| LOC100129636 | 100129636 | 6 | 29036222 | 29076739 | 6p22.1 | - | uncharacterized LOC100129636 | 2.59E-14 |
| OR2W1 | 26692 | 6 | 29045174 | 29044212 | 6p22.1 | - | olfactory receptor, family 2, subfamily W, member 1 | 2.69E-14 |
| TRIM27 | 5987 | 6 | 28923990 | 28903001 | 6p22 | 602165 | tripartite motif containing 27 | 4.48E-14 |
| DDX6 | 1656 | 11 | 118791262 | 118747762 | 11q23.3 | 600326 | DEAD (Asp-Glu-Ala-Asp) box helicase 6 | 1.88E-13 |
| SNORD32B | 692092 | 6 | 29582251 | 29582327 | 6p22.1 | - | small nucleolar RNA, C/D box 32B | 5.3E-13 |
| LINC01185 | 400957 | 2 | 60881313 | 60847759 | 2p16.1 | - | long intergenic non-protein coding RNA 1185 | 1.05E-12 |
| ZSCAN23 | 222696 | 6 | 28443501 | 28431595 | 6p22.1 | - | zinc finger and SCAN domain containing 23 | 3.52E-12 |
| PUS10 | 150962 | 2 | 61018229 | 60940412 | 2p16.1 | 612787 | pseudouridylate synthase 10 | 2.6E-11 |
| LINC01104 | 150577 | 2 | 100208253 | 100251483 | 2q11.2 | - | long intergenic non-protein coding RNA 1104 | 1.57E-10 |
| BAK1 | 578 | 6 | 33580295 | 33572545 | 6p21.3 | 600516 | BCL2-antagonist/killer 1 | 1.78E-10 |
| OR2J3 | 442186 | 6 | 29111809 | 29112883 | 6p22.1 | 615016 | olfactory receptor, family 2, subfamily J, member 3 | 2.42E-10 |
| OR2J2 | 26707 | 6 | 29173533 | 29174573 | 6p22.2-p21.31 | - | olfactory receptor, family 2, subfamily J, member 2 | 2.79E-10 |
| OR12D3 | 81797 | 6 | 29375290 | 29373422 | 6p22.1 | - | olfactory receptor, family 12, subfamily D, member 3 | 2.81E-10 |
| TPI1P2 | 286016 | 7 | 129055222 | 129057238 | 7q32.1 | - | triosephosphate isomerase 1 pseudogene 2 | 2.81E-10 |
| OR2B3 | 442184 | 6 | 29087312 | 29086207 | 6p22.1 | - | olfactory receptor, family 2, subfamily B, member 3 | 4.59E-10 |
| HIST1H2BF | 8343 | 6 | 26199558 | 26199987 | 6p22.1 | 602804 | histone cluster 1, H2bf | 5.06E-10 |
| HIST1H2AD | 3013 | 6 | 26199292 | 26198783 | 6p21.3 | 602792 | histone cluster 1, H2ad | 5.56E-10 |
| HIST1H3D | 8351 | 6 | 26199292 | 26196783 | 6p22.1 | 602811 | histone cluster 1, H3d | 7.06E-10 |
| NCOA5 | 57727 | 20 | 46089940 | 46060984 | 20q12-q13.12 | - | nuclear receptor coactivator 5 | 3.85E-09 |
| MIR4305 | 100422940 | 13 | 39664134 | 39664033 | - | - | microRNA 4305 | 6.87E-09 |
| FAM213B | 127281 | 1 | 2586459 | 2591468 | 1p36.32 | - | family with sequence similarity 213, member B | 9.98E-09 |
| SLC12A5 | 57468 | 20 | 46021689 | 46060149 | 20q13.12 | 606726 | solute carrier family 12 (potassium/chloride transporter), member 5 | 1.06E-08 |
| FAM205A | 259308 | 9 | 34729537 | 34723052 | 9p12 | - | family with sequence similarity 205, member A | 2.10E-08 |
| MMEL1 | 79258 | 1 | 2633041 | 2590641 | 1p36 | - | membrane metallo-endopeptidase-like 1 | 2.33E-08 |
| TREH | 11181 | 11 | 118679671 | 118658231 | 11q23.3 | 275360 | trehalase (brush-border membrane glycoprotein) | 4.20E-08 |
| GSDMB | 55876 | 17 | 39919376 | 39904594 | 17q21.1 | 611221 | gasdermin B | 7.56E-08 |
| C1orf122 | 127687 | 1 | 37807800 | 37809453 | 1p34.3 | - | chromosome 1 open reading frame 122 | 1.52E-07 |
| YRDC | 79693 | 1 | 37808192 | 37802941 | 1p34.3 | 612276 | yrdC N6-threonylcarbamoyltransferase domain containing | 1.52E-07 |
| GRB7 | 2886 | 17 | 39737908 | 39747284 | 17q12 | 601522 | growth factor receptor bound protein 7 | 1.74E-07 |
| LOC100133445 | - | - | - | - | - | - | uncharacterized LOC100133445 | 1.98E-07 |
| TNFRSF14 | 8764 | 1 | 2555766 | 2565621 | 1p36.32 | 602746 | TNF receptor superfamily member 14 | 1.98E-07 |
| OR5V1 | 81696 | 6 | 29457066 | 29355210 | 6p22.1 | - | olfactory receptor family 5 subfamily V member 1 | 2.86E-07 |
| LOC115110 | 115110 | 1 | 2557010 | 2549919 | 1p36.32 | - | uncharacterized LOC115110 | 2.97E-07 |
| MTF1 | 4520 | 1 | 37859623 | 37809566 | 1p34.3 | 600172 | metal regulatory transcription factor 1 | 3.39E-07 |
| PHLDB1 | 23187 | 11 | 118606295 | 118658037 | 11q23.3 | 612834 | pleckstrin homology like domain family B member 1 | 4.27E-07 |
| IKZF3 | 22806 | 17 | 39864187 | 39757714 | 17q12-21.1 | 606221 | IKAROS family zinc finger 3 | 4.27E-07 |
| HMGN4 | 10473 | 6 | 26538343 | 26546936 | 6p22.2 | - | high mobility group nucleosomal binding domain 4 | 6.11E-07 |
| SUOX | 6821 | 12 | 55996775 | 56005524 | 12q13.2 | 606887 | sulfite oxidase | 7.10E-07 |
| ICAM3 | 3385 | 19 | 10339833 | 10333775 | 19p13.2 | 146631 | intercellular adhesion molecule 3 | 8.08E-07 |
| RAB5B | 5869 | 12 | 55973912 | 55996682 | 12q13.2 | 179514 | RAB5B, member RAS oncogene family | 9.04E-07 |
| MANEAL | 149175 | 1 | 37793815 | 37801605 | 1p34.3 | - | mannosidase endo-alpha like | 1.06E-06 |
| TTC34 | 100287898 | 1 | 2789736 | 2636987 | 1p36.32 | - | tetratricopeptide repeat domain 34 | 1.32E-06 |
| AGAP2-AS1 | 100130776 | 12 | 57726239 | 57728355 | 12q14.1 | - | AGAP2 antisense RNA 1 | 1.38E-06 |
| GATA3-AS1 | 399717 | 10 | 8053483 | 8050449 | 10p14 | - | GATA3 antisense RNA 1 | 1.60E-06 |
| RAVER1 | 125950 | 19 | 10333637 | 10316211 | 19p13.2 | 609950 | ribonucleoprotein, PTB binding 1 | 1.60E-06 |
| ORMDL3 | 94103 | 17 | 39927803 | 39921040 | 17q21.1 | 610075 | ORMDL sphingolipid biosynthesis regulator 3 | 1.87E-06 |
| TMPRSS3 | 64699 | 21 | 42396845 | 42371886 | 21q22.3 | 605511 | transmembrane protease, serine 3 | 1.92E-06 |
| LINC01623 | 401242 | 6 | 28863676 | 28859624 | 6p22.1 | - | long intergenic non-protein coding RNA 1623 | 2.16E-06 |

Note:

‘Chr’: Chromosome, ‘-‘: not available, ‘Start’ and ‘stop’: Genomic Location
