# Supplementary material for: Gene-Based Genome-Wide Association Analysis in European and Asian Populations Identified Novel Genes for Rheumatoid Arthritis
Source: PLoS One. 2016 Nov 29;11(11):e0167212. doi: 10.1371/journal.pone.0167212 (PMC5127563; doi:10.1371/journal.pone.0167212)
Supplement: S3 Table — Note: ‘Chr’: Chromosome, ‘-‘: not available, ‘Start’ and ‘stop’: Genomic Location (DOCX) [file pone.0167212.s005.docx]

**Table S3. The 74 ‘Asian-specific’ RA-associated genes newly detected by gene-based association study**

| **Gene Symbol** | **ID** | **Chr** | **Start** | **Stop** | **Map** | **OMIM** | **Description** | **P value** |
| --- | --- | --- | --- | --- | --- | --- | --- | --- |
| OR2H1 | 26716 | 6 | 29458359 | 29464321 | 6p21.32 | - | olfactory receptor, family 2, subfamily H, member 1 | 1.76E-20 |
| FGFR1OP | 11116 | 6 | 167000000 | 167000000 | 6q27 | 605392 | FGFR1 oncogene partner | 3.41E-14 |
| GPX6 | 257202 | 6 | 28515792 | 28503295 | 6p22.1 | 607913 | glutathione peroxidase 6 (olfactory) | 1.82E-13 |
| SCGN | 10590 | 6 | 25652200 | 25701782 | 6p22.3-p22.1 | 609202 | secretagogin, EF-hand calcium binding protein | 8.1E-13 |
| MIR3939 | 100500857 | 6 | 167000000 | 167000000 | 6q27 | - | microRNA 3939 | 1.47E-12 |
| TRIM38 | 10475 | 6 | 25962688 | 25987328 | 6p21.3 | - | tripartite motif containing 38 | 1.93E-12 |
| TCTE1 | 202500 | 6 | 44297720 | 44280159 | 6p21.1 | 186975 | t-complex-associated-testis-expressed 1 | 4.33E-12 |
| RNASET2 | 8635 | 6 | 167000000 | 167000000 | 6q27 | 612944 | ribonuclease T2 | 6.73E-12 |
| MIR3143 | 100422934 | 6 | 27147625 | 27147687 | 6p22.1 | - | microRNA 3143 | 3.12E-11 |
| HIST1H2AH | 85235 | 6 | 27147081 | 27147561 | 6p21.33 | 615013 | histone cluster 1, H2ah | 3.26E-11 |
| HIST1H2BK | 85236 | 6 | 27146857 | 27138292 | 6p21.33 | 615045 | histone cluster 1, H2bk | 3.35E-11 |
| LOC100270746 | 100270746 | 6 | 27020305 | 27019365 | 6p22.2 | - | uncharacterized LOC100270746 | 8.93E-11 |
| POM121L2 | 94026 | 6 | 27312231 | 27309062 | 6p22.1 | - | POM121 transmembrane nucleoporin-like 2 | 8.99E-11 |
| BTN2A3P | 54718 | 6 | 26421390 | 26430587 | 6p22.1 | 613592 | butyrophilin, subfamily 2, member A3, pseudogene | 1.45E-10 |
| PRSS16 | 10279 | 6 | 27247722 | 27256619 | 6p21 | 607169 | protease, serine, 16 (thymus) | 1.68E-10 |
| LOC285819 | 285819 | 6 | 26482508 | 26471943 | 6p22.1 | - | uncharacterized LOC285819 | 1.77E-10 |
| HIST1H2AC | 8334 | 6 | 26124144 | 26124689 | 6p22.1 | 602794 | histone cluster 1, H2ac | 2.26E-10 |
| HIST1H2BC | 8347 | 6 | 26123903 | 26123466 | 6p22.1 | 602847 | histone cluster 1, H2bc | 2.26E-10 |
| AARS2 | 57505 | 6 | 44313356 | 44298725 | 6p21.1 | 612035 | alanyl-tRNA synthetase 2, mitochondrial | 2.35E-10 |
| BTN2A1 | 11120 | 6 | 26457903 | 26476620 | 6p22.1 | 613590 | butyrophilin, subfamily 2, member A1 | 2.48E-10 |
| HFE | 3077 | 6 | 26087280 | 26096116 | 6p21.3 | 613609 | hemochromatosis | 2.92E-10 |
| BTN2A2 | 10385 | 6 | 26382869 | 26394873 | 6p22.1 | 613591 | butyrophilin, subfamily 2, member A2 | 3.76E-10 |
| LINC00240 | 100133205 | 6 | 26956992 | 27023973 | 6p22.2 | - | long intergenic non-protein coding RNA 240 | 4.05E-10 |
| ZNF322 | 79692 | 6 | 26659751 | 26634382 | 6p22.1 | 610847 | zinc finger protein 322 | 4.58E-10 |
| SLC17A3 | 10786 | 6 | 25874242 | 25845099 | 6p21.3 | 611034 | solute carrier family 17 (organic anion transporter), member 3 | 6.21E-10 |
| ABT1 | 29777 | 6 | 26596942 | 26600049 | 6p22.2 | - | activator of basal transcription 1 | 7.66E-10 |
| HIST1H4A | 8359 | 6 | 26021678 | 26022049 | 6p22.1 | 602822 | histone cluster 1, H4a | 8.47E-10 |
| SLC17A1 | 6568 | 6 | 25832058 | 25782896 | 6p22.2 | 182308 | solute carrier family 17 (organic anion transporter), member 1 | 8.81E-10 |
| HIST1H3C | 8352 | 6 | 26045410 | 26045868 | 6p22.1 | 602812 | histone cluster 1, H3c | 9.07E-10 |
| HIST1H2AB | 8335 | 6 | 26033567 | 26033091 | 6p22.1 | 602795 | histone cluster 1, H2ab | 9.96E-10 |
| HIST1H2BB | 3018 | 6 | 26043656 | 26043226 | 6p21.3 | 602803 | histone cluster 1, H2bb | 1.06E-09 |
| HIST1H1A | 3024 | 6 | 26017811 | 26017031 | 6p21.3 | 142709 | histone cluster 1, H1a | 1.12E-09 |
| HIST1H3A | 8350 | 6 | 26020489 | 26020957 | 6p22.1 | 602810 | histone cluster 1, H3a | 1.12E-09 |
| BTN3A2 | 11118 | 6 | 26365158 | 26378319 | 6p22.1 | 613594 | butyrophilin, subfamily 3, member A2 | 1.49E-09 |
| HIST1H1E | 3008 | 6 | 26156330 | 26157114 | 6p21.3 | 142220 | histone cluster 1, H1e | 1.56E-09 |
| HIST1H2AA | 221613 | 6 | 25726561 | 25726062 | 6p22.2 | 613499 | histone cluster 1, H2aa | 1.67E-09 |
| HIST1H2BA | 255626 | 6 | 25726908 | 25727344 | 6p22.2 | 609904 | histone cluster 1, H2ba | 1.67E-09 |
| BTN1A1 | 696 | 6 | 26500348 | 26510424 | 6p22.1 | 601610 | butyrophilin, subfamily 1, member A1 | 1.83E-09 |
| HIST1H1C | 3006 | 6 | 26056470 | 26055739 | 6p21.3 | 142710 | histone cluster 1, H1c | 1.93E-09 |
| HIST1H3B | 8358 | 6 | 26032059 | 26031588 | 6p22.1 | 602819 | histone cluster 1, H3b | 2.07E-09 |
| HIST1H4B | 8366 | 6 | 26027251 | 26026895 | 6p22.1 | 602829 | histone cluster 1, H4b | 2.07E-09 |
| HIST1H2BD | 3017 | 6 | 26157842 | 26171348 | 6p21.3 | 602799 | histone cluster 1, H2bd | 2.71E-09 |
| SLC17A2 | 10246 | 6 | 25930725 | 25912753 | 6p21.3 | 611049 | solute carrier family 17, member 2 | 2.89E-09 |
| VN1R10P | 387316 | 6 | 27324760 | 27325962 | 6p22.1 | - | vomeronasal 1 receptor 10 pseudogene | 3.34E-09 |
| SLC17A4 | 10050 | 6 | 25754698 | 25781174 | 6p22.2 | 604216 | solute carrier family 17, member 4 | 7.04E-09 |
| LRRC16A | 55604 | 6 | 25279427 | 25620529 | 6p22.2 | 609593 | leucine rich repeat containing 16A | 8.26E-09 |
| HIST1H2BI | 8346 | 6 | 26272975 | 26273411 | 6p22.1 | 602807 | histone cluster 1, H2bi | 2.98E-08 |
| HIST1H3G | 8355 | 6 | 26271383 | 26270917 | 6p22.1 | 602815 | histone cluster 1, H3g | 2.98E-08 |
| HIST1H4H | 8365 | 6 | 26285498 | 26285125 | 6p22.1 | 602828 | histone cluster 1, H4h | 3.13E-08 |
| GUSBP2 | 387036 | 6 | 26956553 | 26871486 | 6p21 | - | glucuronidase, beta pseudogene 2 | 3.92E-08 |
| HIST1H2BH | 8345 | 6 | 26251650 | 26252074 | 6p22.2 | 602806 | histone cluster 1 H2B family member h | 6.20E-08 |
| HIST1H4G | 8369 | 6 | 26246976 | 26246610 | 6p22.2 | 602832 | histone cluster 1 H4 family member g | 7.42E-08 |
| HIST1H3F | 8968 | 6 | 26250606 | 26250141 | 6p22.2 | 602816 | histone cluster 1 H3 family member f | 7.95E-08 |
| HIST1H4F | 8361 | 6 | 26240425 | 26240792 | 6p22.2 | 602824 | histone cluster 1 H4 family member f | 1.15E-07 |
| HIST1H1D | 3007 | 6 | 26234987 | 26234211 | 6p22.2 | 142210 | histone cluster 1 H1 family member d | 1.36E-07 |
| HIST1H2AJ | 8331 | 6 | 27814739 | 27814301 | 6p22.1 | 602791 | histone cluster 1 H2A family member j | 1.92E-07 |
| HIST1H3H | 8357 | 6 | 27810063 | 27810535 | 6p22.1 | 602818 | histone cluster 1 H3 family member h | 1.98E-07 |
| HIST1H2AE | 3012 | 6 | 26216919 | 26217482 | 6p22.2 | 602786 | histone cluster 1 H2A family member e | 2.38E-07 |
| HIST1H2APS1 | 387319 | 6 | 25732432 | 25732860 | 6p22.2 | - | histone cluster 1 H2A pseudogene 1 | 2.45E-07 |
| HIST1H2BN | 8341 | 6 | 27838661 | 27839109 | 6p22.1 | 602801 | histone cluster 1 H2B family member n | 2.64E-07 |
| HIST1H2BG | 8339 | 6 | 26216643 | 26216199 | 6p22.2 | 602798 | histone cluster 1 H2B family member g | 2.77E-07 |
| HIST1H2AK | 8330 | 6 | 27838338 | 27837879 | 6p22.1 | 602788 | histone cluster 1 H2A family member k | 3.27E-07 |
| HIST1H2BM | 8342 | 6 | 27815043 | 27815488 | 6p22.1 | 602802 | histone cluster 1 H2B family member m | 4.23E-07 |
| HIST1H2BO | 8348 | 6 | 27893424 | 27893890 | 6p22.1 | 602808 | histone cluster 1 H2B family member o | 8.40E-07 |
| HIST1H4J | 8363 | 6 | 27824124 | 27824479 | 6p22.1 | 602826 | histone cluster 1 H4 family member j | 9.21E-07 |
| MAPK14 | 1432 | 6 | 36027634 | 36122963 | 6p21.31 | 600289 | mitogen-activated protein kinase 14 | 9.42E-07 |
| SLC26A8 | 116369 | 6 | 36024854 | 35943513 | 6p21.31 | 608480 | solute carrier family 26 member 8 | 1.04E-06 |
| HIST1H2AM | 8336 | 6 | 27893184 | 27892698 | 6p22.1 | 602796 | histone cluster 1 H2A family member m | 1.08E-06 |
| HIST1H3J | 8356 | 6 | 27890791 | 27890314 | 6p22.1 | 602817 | histone cluster 1 H3 family member j | 1.08E-06 |
| HIST1H4K | 8362 | 6 | 27831526 | 27831173 | 6p22.1 | 602825 | histone cluster 1 H4 family member k | 1.12E-06 |
| HIST1H2AL | 8332 | 6 | 27865328 | 27865797 | 6p22.1 | 602793 | histone cluster 1 H2A family member l | 1.26E-06 |
| MAPK13 | 5603 | 6 | 36130483 | 36144523 | 6p21.31 | 602899 | mitogen-activated protein kinase 13 | 1.40E-06 |
| HIST1H2AI | 8329 | 6 | 27808198 | 27808666 | 6p22.1 | 602787 | histone cluster 1 H2A family member i | 1.41E-06 |
| HIST1H2BL | 8340 | 6 | 27807930 | 27807478 | 6p22.1 | 602800 | histone cluster 1 H2B family member l | 1.41E-06 |

Note:

‘Chr’: Chromosome, ‘-‘: not available, ‘Start’ and ‘stop’: Genomic Location
