# Supplementary material for: Gene-Based Genome-Wide Association Analysis in European and Asian Populations Identified Novel Genes for Rheumatoid Arthritis
Source: PLoS One. 2016 Nov 29;11(11):e0167212. doi: 10.1371/journal.pone.0167212 (PMC5127563; doi:10.1371/journal.pone.0167212)
Supplement: S4 Table — Note: RA: rheumatoid arthritis; HC: health controls; PBMC: peripheral blood mononuclear cell; GSE number: Gene Expression Omnibus, http://www.ncbi.nlm.nih.gov/geo/; ★t overlapped genes; ◆o European-specific genes; ●u Asian-specific genes. We only listed the most significant expression results of probes if one gene has multiple detected probes. (DOCX) [file pone.0167212.s006.docx]

**Table S4. Differential expression analyses for the “novel” RA-associated genes identified by gene-based study**

| **GSE Number** | | GSE55235 | | | | | GSE55457 | | | | GSE15573 | | | | | GSE17755 | | | | | |
| --- | --- | --- | --- | --- | --- | --- | --- | --- | --- | --- | --- | --- | --- | --- | --- | --- | --- | --- | --- | --- | --- |
| **Ethnicity of subjects** | | Caucasians | | | | | Caucasians | | | | French Caucasians | | | | | Asians | | | | | |
| **Sample size (RA:HC)** | | 10:10 | | | | | 13:10 | | | | 18:15 | | | | | 112:45 | | | | | |
| **Tissue** | | synovial tissue | | | | | synovial tissue | | | | PBMCs | | | | | PBMCs | | | | | |
| **Platform** | | Affymetrix Human Genome U133A Array | | | | | Affymetrix Human Genome U133A Array | | | | Illumina human-6 v2.0 expression beadchip | | | | | Hitachisoft AceGene Human Oligo Chip 30K 1 Chip Version | | | | | |
| **Ref.** | | [23] | | | | | [23] | | | | [24] | | | | | [25] | | | | | |
| **GSE55235** | | | | | **GSE55457** | | | | | **GSE15573** | | | | | | | **GSE17755** | | | | |
| Probe ID | Gene Symbol | | RA/HC | T test  P-value | Probe ID | Gene Symbol | | RA/HC | T test  P-value | Probe ID | | Gene Symbol | RA/HC | | T test  P-value | | Probe ID | | Gene Symbol | RA/HC | T test  P-value |
| 200045_at | ABCF1★ | | 0.71 | 4.28E-03 | 214911_s_at | BRD2★ | | 0.72 | 1.38E-03 | ILMN_1763875 | | ABCF1★ | 0.78 | | 5.16E-04 | | AGhsB210821 | AARS2● | | 3.62 | 6.62E-04 |
| 203728_at | BAK1◆ | | 1.70 | 1.53E-04 | 211256_x_at | BTN2A1● | | 1.42 | 6.38E-05 | ILMN_1669377 | | AP4B1◆ | 0.78 | | 1.83E-05 | | AGhsA101220 | ABCF1★ | | -1.47 | 2.20E-02 |
| 214911_s_at | BRD2★ | | 0.58 | 1.44E-04 | 205298_s_at | BTN2A2● | | 1.85 | 8.07E-05 | ILMN_1805990 | | BAK1◆ | 1.14 | | 3.42E-02 | | AGhsA251317 | AP4B1◆ | | 36.93 | 1.58E-09 |
| 211256_x_at | BTN2A1● | | 1.40 | 9.24E-03 | 209770_at | BTN3A1★ | | 1.89 | 7.95E-05 | ILMN_1758918 | | BRD2★ | 0.88 | | 2.31E-03 | | AGhsB030601 | B3GALT4◆ | | -3.33 | 5.17E-03 |
| 205298_s_at | BTN2A2● | | 2.02 | 6.77E-05 | 209846_s_at | BTN3A2● | | 2.51 | 1.29E-03 | ILMN_1700067 | | BTN3A3★ | 0.76 | | 8.77E-04 | | AGhsA101508 | BAK1◆ | | -1.26 | 3.57E-02 |
| 209770_at | BTN3A1★ | | 2.06 | 6.78E-06 | 38241_at | BTN3A3★ | | 1.93 | 3.45E-04 | ILMN_1786273 | | C1ORF122◆ | 1.13 | | 1.23E-02 | | AGhsA210810 | BRD2★ | | 6.60 | 2.50E-13 |
| 212613_at | BTN3A2● | | 3.56 | 5.40E-05 | 206193_s_at | CDSN★ | | 0.74 | 9.63E-03 | ILMN_1813236 | | C6ORF136★ | 0.83 | | 8.18E-06 | | AGhsA210322 | BTN1A1● | | 2.15 | 4.86E-02 |
| 204821_at | BTN3A3★ | | 4.74 | 7.60E-09 | 219490_s_at | DCLRE1B◆ | | 1.44 | 1.93E-02 | ILMN_1655382 | | DCLRE1B◆ | 0.95 | | 2.45E-02 | | AGhsA041509 | BTN2A2● | | 4.29 | 2.70E-03 |
| 221488_s_at | CUTA◆ | | 1.30 | 1.63E-02 | 210142_x_at | FLOT1★ | | 1.42 | 5.95E-05 | ILMN_1777340 | | DDX6◆ | 1.07 | | 1.46E-02 | | AGhsA051511 | BTN3A3★ | | -114.72 | 4.51E-13 |
| 203694_s_at | DHX16★ | | 0.80 | 3.75E-02 | 211332_x_at | HFE● | | 0.80 | 6.61E-03 | ILMN_1661439 | | FLOT1★ | 1.17 | | 1.47E-02 | | AGhsC040510 | C6orf136★ | | -0.97 | 1.50E-05 |
| 205588_s_at | FGFR1OP● | | 1.72 | 1.38E-03 | 212293_at | HIPK1◆ | | 0.78 | 3.63E-03 | ILMN_1814611 | | HCG9★ | 1.08 | | 2.14E-02 | | AGhsA041422 | CDSN★ | | 0.24 | 4.21E-11 |
| 211330_s_at | HFE● | | 1.76 | 9.48E-05 | 214469_at | HIST1H2AE● | | 2.36 | 3.62E-02 | ILMN_1721127 | | HIST1H3D◆ | 1.13 | | 5.93E-03 | | AGhsA211111 | CUTA◆ | | -3.29 | 1.89E-09 |
| 208547_at | HIST1H2BB● | | 0.38 | 4.20E-03 | 214542_x_at | HIST1H2AI● | | 0.42 | 2.16E-02 | ILMN_1695311 | | HLA-DMA★ | 0.72 | | 6.50E-04 | | AGhsA041611 | DAXX◆ | | -2.86 | 1.31E-09 |
| 214516_at | HIST1H4B● | | 0.60 | 3.02E-02 | 213932_x_at | HLA-A★ | | 0.82 | 5.32E-03 | ILMN_1673711 | | HSP90AB1★ | 0.83 | | 1.68E-02 | | AGhsB131413 | DDX6◆ | | -80.11 | 4.83E-04 |
| 217478_s_at | HLA-DMA★ | | 4.46 | 1.60E-07 | 217478_s_at | HLA-DMA★ | | 1.46 | 2.05E-02 | ILMN_1682717 | | IER3★ | 1.40 | | 1.28E-02 | | AGhsB201221 | FGFR1OP● | | 14.99 | 2.52E-03 |
| 221875_x_at | HLA-F★ | | 1.64 | 4.39E-04 | 221978_at | HLA-F★ | | 1.59 | 2.93E-03 | ILMN_1765451 | | MAGI3◆ | 1.04 | | 2.47E-02 | | AGhsA201616 | FLOT1★ | | 0.38 | 4.20E-02 |
| 210514_x_at | HLA-G★ | | 1.70 | 3.19E-03 | 211529_x_at | HLA-G★ | | 1.25 | 3.95E-02 | ILMN_1749327 | | MAPK13● | 1.16 | | 3.52E-02 | | AGhsB021013 | HCG4★ | | 0.57 | 1.11E-05 |
| 200064_at | HSP90AB1★ | | 0.52 | 6.62E-05 | 202579_x_at | HMGN4◆ | | 1.50 | 1.00E-06 | ILMN_1763828 | | MTF1◆ | 1.33 | | 2.30E-03 | | AGhsB040307 | HFE● | | 0.60 | 4.55E-06 |
| 204949_at | ICAM3◆ | | 4.82 | 1.01E-06 | 201631_s_at | IER3★ | | 0.37 | 1.35E-02 | ILMN_1803464 | | PHTF1◆ | 1.19 | | 1.67E-02 | | AGhsB140602 | HIPK1◆ | | -6.64 | 4.11E-05 |
| 201631_s_at | IER3★ | | 0.18 | 3.18E-02 | 210449_x_at | MAPK14● | | 0.60 | 9.79E-04 | ILMN_1679060 | | POU5F1★ | 1.09 | | 3.46E-02 | | AGhsA210403 | HIST1H1C● | | -1.38 | 9.18E-06 |
| 210058_at | MAPK13● | | 1.92 | 7.37E-06 | 221339_at | OR10C1★ | | 0.72 | 2.08E-02 | ILMN_1750167 | | PRR3★ | 0.94 | | 4.28E-02 | | AGhsC061224 | HIST1H1D● | | 2.53 | 5.47E-05 |
| 210191_s_at | PHTF1◆ | | 1.48 | 3.38E-04 | 221431_s_at | OR12D3◆ | | 0.61 | 2.38E-02 | ILMN_1725719 | | RAVER1◆ | 0.87 | | 2.22E-02 | | AGhsA210324 | HIST1H1E● | | 7.68 | 5.62E-12 |
| 217984_at | RNASET2● | | 2.31 | 8.74E-08 | 222029_x_at | PFDN6◆ | | 0.65 | 2.01E-03 | ILMN_1759159 | | RGL2◆ | 1.21 | | 8.46E-03 | | AGhsB260608 | HIST1H2AC● | | -1.88 | 1.66E-22 |
| 204067_at | SUOX◆ | | 1.39 | 3.99E-02 | 205702_at | PHTF1◆ | | 1.29 | 4.91E-03 | ILMN_1753534 | | RPS18◆ | 1.78 | | 4.42E-02 | | AGhsB081521 | HIST1H2AD◆ | | -24.60 | 2.75E-08 |
| 209354_at | TNFRSF14◆ | | 1.53 | 1.80E-02 | 201702_s_at | PPP1R10★ | | 0.46 | 2.00E-02 | ILMN_1773757 | | SLC26A8● | 1.24 | | 2.24E-03 | | AGhsB081221 | HIST1H2AE● | | -1.74 | 1.06E-05 |
| 212116_at | TRIM27◆ | | 1.52 | 3.80E-04 | 201500_s_at | PPP1R11★ | | 1.20 | 4.58E-03 | ILMN_1803745 | | SUOX◆ | 0.89 | | 4.55E-03 | | AGhsB211212 | HIST1H2BK● | | -18.88 | 3.29E-07 |
| 203567_s_at | TRIM38● | | 1.47 | 1.42E-02 | 217983_s_at | RNASET2● | | 1.43 | 4.50E-02 | ILMN_1697409 | | TNFRSF14◆ | 0.89 | | 2.91E-03 | | AGhsB081621 | HIST1H3D◆ | | -6.30 | 2.90E-04 |
| 212320_at | TUBB★ | | 1.34 | 3.85E-02 | 201049_s_at | RPS18◆ | | 0.74 | 1.36E-04 | ILMN_1704750 | | TUBB★ | 0.80 | | 1.10E-04 | | AGhsB080422 | HIST1H3F● | | -0.80 | 1.46E-05 |
| 218647_s_at | YRDC◆ | | 0.80 | 4.27E-02 | 213676_at | TMEM151B★ | | 0.67 | 1.10E-02 | ILMN_1809566 | | ZSCAN16★ | 1.10 | | 4.11E-02 | | AGhsB080522 | HIST1H4F● | | -0.51 | 4.48E-06 |
| 213625_at | ZKSCAN4★ | | 1.79 | 4.68E-04 | 209354_at | TNFRSF14◆ | | 1.66 | 4.35E-05 |  | |  | |  |  | | AGhsB181310 | HLA-A★ | | 2.25 | 2.82E-07 |
|  |  | |  |  | 212118_at | TRIM27◆ | | 1.15 | 4.50E-02 |  | |  | |  |  | | AGhsC061407 | HLA-DMA★ | | 7.55 | 1.38E-16 |
|  |  | |  |  | 209196_at | WDR46◆ | | 0.60 | 3.69E-03 |  | |  | |  |  | | AGhsB120817 | HLA-F★ | | 2.39 | 2.61E-02 |
|  |  | |  |  | 211773_s_at | ZKSCAN3★ | | 0.78 | 2.80E-02 |  | |  | |  |  | | AGhsB141508 | HLA-G★ | | 1.90 | 2.24E-05 |
|  |  | |  |  | 213625_at | ZKSCAN4★ | | 1.49 | 9.92E-03 |  | |  | |  |  | | AGhsB260717 | HMGN4◆ | | 4.12 | 1.71E-21 |
|  |  | |  |  |  |  | |  |  |  | |  | |  |  | | AGhsA210114 | HSP90AB1★ | | 22.87 | 7.70E-33 |
|  |  | |  |  |  |  | |  |  |  | |  | |  |  | | AGhsA140902 | ICAM3◆ | | 28.41 | 6.92E-06 |
|  |  | |  |  |  |  | |  |  |  | |  | |  |  | | AGhsB080615 | KIFC1★ | | 9.37 | 1.11E-09 |
|  |  | |  |  |  |  | |  |  |  | |  | |  |  | | AGhsB140702 | MAGI3◆ | | -3.60 | 1.00E-03 |
|  |  | |  |  |  |  | |  |  |  | |  | |  |  | | AGhsA211204 | MAPK13● | | 23.66 | 1.18E-06 |
|  |  | |  |  |  |  | |  |  |  | |  | |  |  | | AGhsA211203 | MAPK14● | | 3.06 | 1.66E-03 |
|  |  | |  |  |  |  | |  |  |  | |  | |  |  | | AGhsB141212 | MAS1L★ | | 3.08 | 1.06E-08 |
|  |  | |  |  |  |  | |  |  |  | |  | |  |  | | AGhsA211009 | MLN★ | | 3.29 | 7.33E-03 |
|  |  | |  |  |  |  | |  |  |  | |  | |  |  | | AGhsA201617 | MRPS18B★ | | 2.66 | 2.29E-09 |
|  |  | |  |  |  |  | |  |  |  | |  | |  |  | | AGhsB231616 | NCOA5◆ | | -10.65 | 1.72E-05 |
|  |  | |  |  |  |  | |  |  |  | |  | |  |  | | AGhsA251316 | OLFML3◆ | | 4.50 | 1.18E-07 |
|  |  | |  |  |  |  | |  |  |  | |  | |  |  | | AGhsA210814 | OR11A1★ | | 0.46 | 1.50E-07 |
|  |  | |  |  |  |  | |  |  |  | |  | |  |  | | AGhsA210813 | OR12D2★ | | 4.36 | 5.86E-05 |
|  |  | |  |  |  |  | |  |  |  | |  | |  |  | | AGhsB260404 | OR2B3◆ | | 81.60 | 1.38E-04 |
|  |  | |  |  |  |  | |  |  |  | |  | |  |  | | AGhsB021116 | OR2H2◆ | | 0.41 | 1.36E-03 |
|  |  | |  |  |  |  | |  |  |  | |  | |  |  | | AGhsB260604 | OR2J2◆ | | -0.27 | 4.64E-06 |
|  |  | |  |  |  |  | |  |  |  | |  | |  |  | | AGhsB260804 | OR2W1◆ | | 7.01 | 5.38E-06 |
|  |  | |  |  |  |  | |  |  |  | |  | |  |  | | AGhsB090213 | OR5V1◆ | | 2.48 | 2.90E-03 |
|  |  | |  |  |  |  | |  |  |  | |  | |  |  | | AGhsA150912 | ORMDL3◆ | | -6.27 | 8.72E-13 |
|  |  | |  |  |  |  | |  |  |  | |  | |  |  | | AGhsA211109 | PFDN6◆ | | 1.66 | 8.68E-03 |
|  |  | |  |  |  |  | |  |  |  | |  | |  |  | | AGhsB241015 | PGBD1★ | | 2.24 | 5.55E-03 |
|  |  | |  |  |  |  | |  |  |  | |  | |  |  | | AGhsB080608 | PHLDB1◆ | | 1.98 | 5.56E-03 |
|  |  | |  |  |  |  | |  |  |  | |  | |  |  | | AGhsA251320 | PHTF1◆ | | -2.45 | 6.43E-13 |
|  |  | |  |  |  |  | |  |  |  | |  | |  |  | | AGhsA101221 | POU5F1★ | | -1.21 | 1.24E-03 |
|  |  | |  |  |  |  | |  |  |  | |  | |  |  | | AGhsA201618 | PPP1R10★ | | 3.79 | 5.96E-03 |
|  |  | |  |  |  |  | |  |  |  | |  | |  |  | | AGhsA101219 | PPP1R11★ | | -1.59 | 1.08E-02 |
|  |  | |  |  |  |  | |  |  |  | |  | |  |  | | AGhsC030706 | PRR3★ | | 1.78 | 1.07E-10 |
|  |  | |  |  |  |  | |  |  |  | |  | |  |  | | AGhsC021308 | RAB5B◆ | | -0.81 | 1.06E-24 |
|  |  | |  |  |  |  | |  |  |  | |  | |  |  | | AGhsB061122 | RNASET2● | | -7.28 | 4.89E-05 |
|  |  | |  |  |  |  | |  |  |  | |  | |  |  | | AGhsB260202 | RNF39★ | | -0.47 | 1.18E-24 |
|  |  | |  |  |  |  | |  |  |  | |  | |  |  | | AGhsA011323 | RPS18◆ | | -87.71 | 1.60E-02 |
|  |  | |  |  |  |  | |  |  |  | |  | |  |  | | AGhsA251319 | RSBN1◆ | | -8.50 | 4.92E-03 |
|  |  | |  |  |  |  | |  |  |  | |  | |  |  | | AGhsA030902 | SCGN● | | 3.20 | 1.73E-02 |
|  |  | |  |  |  |  | |  |  |  | |  | |  |  | | AGhsA051419 | SLC17A1● | | 16.77 | 3.23E-04 |
|  |  | |  |  |  |  | |  |  |  | |  | |  |  | | AGhsB250303 | SLC17A3● | | -1.96 | 6.13E-31 |
|  |  | |  |  |  |  | |  |  |  | |  | |  |  | | AGhsB010723 | SLC17A4● | | -1.19 | 1.61E-03 |
|  |  | |  |  |  |  | |  |  |  | |  | |  |  | | AGhsC040213 | SLC26A8● | | -0.80 | 6.93E-03 |
|  |  | |  |  |  |  | |  |  |  | |  | |  |  | | AGhsA171321 | SUOX◆ | | 22.61 | 8.49E-07 |
|  |  | |  |  |  |  | |  |  |  | |  | |  |  | | AGhsB091408 | SYNGAP1★ | | -3.11 | 1.02E-02 |
|  |  | |  |  |  |  | |  |  |  | |  | |  |  | | AGhsA011117 | TMPRSS3◆ | | -4.53 | 4.20E-09 |
|  |  | |  |  |  |  | |  |  |  | |  | |  |  | | AGhsA050713 | TNFRSF14◆ | | 160.43 | 1.26E-11 |
|  |  | |  |  |  |  | |  |  |  | |  | |  |  | | AGhsA210405 | TRIM38● | | 0.21 | 1.16E-16 |
|  |  | |  |  |  |  | |  |  |  | |  | |  |  | | AGhsB241617 | TUBB★ | | -34.40 | 6.19E-05 |
|  |  | |  |  |  |  | |  |  |  | |  | |  |  | | AGhsA070111 | VPS52◆ | | -1.51 | 4.30E-05 |
|  |  | |  |  |  |  | |  |  |  | |  | |  |  | | AGhsB100512 | WDR46◆ | | -0.93 | 3.25E-06 |
|  |  | |  |  |  |  | |  |  |  | |  | |  |  | | AGhsA070812 | YRDC◆ | | 0.32 | 3.79E-02 |
|  |  | |  |  |  |  | |  |  |  | |  | |  |  | | AGhsB111517 | ZKSCAN3★ | | 4.44 | 1.57E-03 |
|  |  | |  |  |  |  | |  |  |  | |  | |  |  | | AGhsA101304 | ZKSCAN4★ | | -8.23 | 8.61E-03 |
|  |  | |  |  |  |  | |  |  |  | |  | |  |  | | AGhsA210310 | ZNF165★ | | 2.37 | 1.55E-02 |
|  |  | |  |  |  |  | |  |  |  | |  | |  |  | | AGhsC070814 | ZNF311◆ | | 7.49 | 1.72E-05 |
|  |  | |  |  |  |  | |  |  |  | |  | |  |  | | AGhsA020618 | ZNRD1★ | | -6.53 | 1.93E-04 |
|  |  | |  |  |  |  | |  |  |  | |  | |  |  | | AGhsB221516 | ZSCAN23◆ | | 11.69 | 8.78E-03 |

Note:

RA: rheumatoid arthritis; HC: health controls; PBMC: peripheral blood mononuclear cell; GSE number: Gene Expression Omnibus, <http://www.ncbi.nlm.nih.gov/geo/>; ★: overlapped genes; ◆:European-specific genes; ●: Asian-specific genes. We only listed the most significant expression results of probes if one gene has multiple detected probes.
