# Supplementary material for: Gene-Based Genome-Wide Association Analysis in European and Asian Populations Identified Novel Genes for Rheumatoid Arthritis
Source: PLoS One. 2016 Nov 29;11(11):e0167212. doi: 10.1371/journal.pone.0167212 (PMC5127563; doi:10.1371/journal.pone.0167212)
Supplement: S5 Table — Note: Functional annotation clustering analysis was performed using DAVID. (DOCX) [file pone.0167212.s007.docx]

**Table S5. Functional annotation clustering analysis for the 221 newly Identified RA-associated Genes.**

| **Category** | **Term** | **Count** | **%** | **P Value** | **Genes** | **Pop Hits** | **Fold Enrichment** | **Bonferroni** |
| --- | --- | --- | --- | --- | --- | --- | --- | --- |
| GOTERM_BP_DIRECT | GO:0006334~nucleosome assembly | 34 | 15.81 | 9.04E-38 | HIST1H4K, DAXX, HIST1H2BO, HIST1H2BM, HIST1H2BN, HIST1H2BK, HIST1H4A, HIST1H2BL, HIST1H4B, HIST1H2BI, HIST1H4E, HIST1H4F, HIST1H4J, HIST1H4H, HIST1H2BA, HIST1H3J, HIST1H2BB, HIST1H1E, HIST1H2BC, BRD2, HIST1H1D, HIST1H2BD, HIST1H1C, HIST1H2BF, HIST1H1A, HIST1H2BG, HIST1H2BH, HIST1H3A, HIST1H3B, HIST1H3C, HIST1H3D, HIST1H3F, HIST1H3G, HIST1H3H | 134 | 26.19 | 5.94E-35 |
| GOTERM_BP_DIRECT | GO:0006325~chromatin organization | 28 | 13.02 | 2.06E-21 | HIST1H4K, HIST1H2BO, HIST1H2BM, HIST1H2BN, HIST1H2BK, HIST1H4A, HIST1H2BL, HIST1H4B, HIST1H2BI, HIST1H4E, HIST1H4F, HIST1H4J, HIST1H4H, HIST1H2BA, HIST1H2BB, HIST1H3J, HIST1H2BC, HIST1H2BD, HIST1H2BF, HIST1H2BG, HIST1H2BH, HIST1H3A, HIST1H3B, HIST1H3C, HIST1H3D, HIST1H3F, HIST1H3G, HIST1H3H | 239 | 12.09 | 1.35E-18 |
| GOTERM_BP_DIRECT | GO:0032200~telomere organization | 15 | 6.98 | 5.84E-22 | HIST1H3J, HIST1H4K, HIST1H4A, HIST1H4B, HIST1H3A, HIST1H3B, HIST1H4E, HIST1H3C, HIST1H4F, HIST1H3D, HIST1H3F, HIST1H3G, HIST1H4J, HIST1H3H, HIST1H4H | 27 | 57.34 | 3.84E-19 |
| GOTERM_BP_DIRECT | GO:0006335~DNA replication-dependent nucleosome assembly |  |  | 1.31E-20 |  | 32 | 48.38 | 8.63E-18 |
| GOTERM_BP_DIRECT | GO:0032776~DNA methylation on cytosine |  |  | 3.81E-20 |  | 34 | 45.54 | 2.50E-17 |
| GOTERM_BP_DIRECT | GO:0051290~protein heterotetramerization |  |  | 2.55E-19 |  | 38 | 40.74 | 1.68E-16 |
| GOTERM_BP_DIRECT | GO:0000183~chromatin silencing at rDNA |  |  | 9.06E-19 |  | 41 | 37.76 | 5.95E-16 |
| GOTERM_BP_DIRECT | GO:0031047~gene silencing by RNA |  |  | 6.43E-12 |  | 117 | 13.23 | 4.22E-09 |
| GOTERM_BP_DIRECT | GO:0045815~positive regulation of gene expression, epigenetic | 16 | 7.44 | 1.77E-17 | HIST1H3J, HIST1H4K, ZNRD1, HIST1H4A, HIST1H4B, HIST1H3A, HIST1H3B, HIST1H4E, HIST1H4F, HIST1H3C, HIST1H3D, HIST1H3F, HIST1H4J, HIST1H3G, HIST1H3H, HIST1H4H | 62 | 26.64 | 1.16E-14 |
| GOTERM_BP_DIRECT | GO:0040029~regulation of gene expression, epigenetic |  |  | 3.91E-13 |  | 117 | 14.12 | 2.57E-10 |
| GOTERM_BP_DIRECT | GO:0045814~negative regulation of gene expression, epigenetic | 17 | 7.91 | 5.81E-16 | HIST1H3J, HIST1H4K, TRIM27, ZNRD1, HIST1H4A, HIST1H4B, HIST1H3A, HIST1H3B, HIST1H4E, HIST1H4F, HIST1H3C, HIST1H3D, HIST1H3F, HIST1H4J, HIST1H3G, HIST1H3H, HIST1H4H | 94 | 18.67 | 3.65E-13 |
| GOTERM_BP_DIRECT | GO:0060968~regulation of gene silencing | 8 | 3.72 | 2.14E-12 | HIST1H3J, HIST1H3A, HIST1H3B, HIST1H3C, HIST1H3D, HIST1H3F, HIST1H3G, HIST1H3H | 11 | 75.07 | 1.41E-09 |
| GOTERM_BP_DIRECT | GO:0006342~chromatin silencing | 10 | 4.65 | 3.00E-10 | HIST1H2AB, HIST1H2AC, HIST1H2AA, HIST1H2AD, HIST1H2AE, HIST1H2AI, HIST1H2AH, HIST1H2AK, HIST1H2AM, HIST1H2AL | 44 | 23.46 | 1.97E-07 |
| GOTERM_BP_DIRECT | GO:0035574~histone H4-K20 demethylation | 7 | 3.26 | 5.35E-09 | HIST1H4K, HIST1H4A, HIST1H4B, HIST1H4E, HIST1H4F, HIST1H4J, HIST1H4H | 16 | 45.16 | 3.52E-06 |
| GOTERM_BP_DIRECT | GO:0045653~negative regulation of megakaryocyte differentiation |  |  | 1.22E-08 |  | 18 | 40.14 | 8.02E-06 |
| GOTERM_BP_DIRECT | GO:0006336~DNA replication-independent nucleosome assembly |  |  | 1.42E-07 |  | 26 | 27.79 | 9.34E-05 |
| GOTERM_BP_DIRECT | GO:0006303~double-strand break repair via nonhomologous end joining | 8 | 3.72 | 2.36E-06 | DCLRE1B, HIST1H4K, HIST1H4A, HIST1H4B, HIST1H4E, HIST1H4F, HIST1H4J, HIST1H4H | 63 | 13.11 | 1.55E-03 |
| GOTERM_BP_DIRECT | GO:0034080~CENP-A containing nucleosome assembly | 7 | 3.26 | 2.85E-06 | HIST1H4K, HIST1H4A, HIST1H4B, HIST1H4E, HIST1H4F, HIST1H4J, HIST1H4H | 42 | 17.20 | 1.87E-03 |
| GOTERM_BP_DIRECT | GO:0000723~telomere maintenance | 8 | 3.72 | 3.24E-06 | DCLRE1B, HIST1H4K, HIST1H4A, HIST1H4B, HIST1H4E, HIST1H4F, HIST1H4J, HIST1H4H | 66 | 12.51 | 2.13E-03 |
| GOTERM_BP_DIRECT | GO:0044341~sodium-dependent phosphate transport | 4 | 1.86 | 4.73E-05 | SLC17A3, SLC17A4, SLC17A1, SLC17A2 | 8 | 51.61 | 3.06E-02 |
| GOTERM_BP_DIRECT | GO:0019731~antibacterial humoral response | 6 | 2.79 | 5.56E-05 | HIST1H2BC, HIST1H2BK, HIST1H2BF, HIST1H2BG, HIST1H2BI, HLA-A | 43 | 14.40 | 3.59E-02 |
| GOTERM_BP_DIRECT | GO:0098792~xenophagy | 8 | 3.72 | 6.93E-05 | HIST1H3J, HIST1H3A, HIST1H3B, HIST1H3C, HIST1H3D, HIST1H3F, HIST1H3G, HIST1H3H | 105 | 7.86 | 4.45E-02 |
| GOTERM_BP_DIRECT | GO:0002227~innate immune response in mucosa | 5 | 2.33 | 7.56E-05 | HIST1H2BC, HIST1H2BK, HIST1H2BF, HIST1H2BG, HIST1H2BI | 24 | 21.50 | 4.85E-02 |
| KEGG_PATHWAY | hsa05322:Systemic lupus erythematosus | 41 | 19.07 | 5.02E-48 | HIST1H2AB, HIST1H2AC, HIST1H4K, HIST1H2AA, HIST1H2AD, HIST1H2AE, HLA-DMA, HIST1H2BO, HIST1H2BM, HIST1H2BN, HIST1H2BK, HIST1H4A, HIST1H4B, HIST1H2BL, HIST1H2BI, HIST1H4E, HIST1H4F, HIST1H4J, HIST1H4G, HIST1H4H, HIST1H2BA, HIST1H3J, HIST1H2BB, HIST1H2BC, HIST1H2BD, HIST1H2BF, HIST1H2BG, HIST1H2BH, HIST1H3A, HIST1H3B, HIST1H2AI, HIST1H2AH, HIST1H3C, HIST1H2AK, HIST1H3D, HIST1H2AJ, HIST1H3F, HIST1H2AM, HIST1H3G, HIST1H2AL, HIST1H3H | 134 | 24.81 | 4.41E-46 |
| KEGG_PATHWAY | hsa05034:Alcoholism | 40 | 18.60 | 6.20E-41 | HIST1H2AB, HIST1H2AC, HIST1H4K, HIST1H2AA, HIST1H2AD, HIST1H2AE, HIST1H2BO, HIST1H2BM, HIST1H2BN, HIST1H2BK, HIST1H4A, HIST1H4B, HIST1H2BL, HIST1H2BI, HIST1H4E, HIST1H4F, HIST1H4J, HIST1H4G, HIST1H4H, HIST1H2BA, HIST1H3J, HIST1H2BB, HIST1H2BC, HIST1H2BD, HIST1H2BF, HIST1H2BG, HIST1H2BH, HIST1H3A, HIST1H3B, HIST1H2AI, HIST1H2AH, HIST1H3C, HIST1H2AK, HIST1H3D, HIST1H2AJ, HIST1H3F, HIST1H2AM, HIST1H3G, HIST1H2AL, HIST1H3H | 177 | 18.32 | 5.45E-39 |
| KEGG_PATHWAY | hsa05203:Viral carcinogenesis | 26 | 12.09 | 4.98E-19 | HIST1H2BA, HIST1H2BB, HIST1H2BC, HIST1H4K, HIST1H2BD, HIST1H2BF, HIST1H2BG, HIST1H2BH, HLA-A, HLA-G, HLA-F, HIST1H2BO, BAK1, HIST1H2BM, HIST1H2BN, HIST1H2BK, HIST1H4A, MRPS18B, HIST1H4B, HIST1H2BL, HIST1H2BI, HIST1H4E, HIST1H4F, HIST1H4J, HIST1H4G, HIST1H4H | 205 | 10.28 | 4.38E-17 |

Note: Functional annotation clustering analysis was performed using DAVID.
