# Supplementary material for: Gene-Based Genome-Wide Association Analysis in European and Asian Populations Identified Novel Genes for Rheumatoid Arthritis
Source: PLoS One. 2016 Nov 29;11(11):e0167212. doi: 10.1371/journal.pone.0167212 (PMC5127563; doi:10.1371/journal.pone.0167212)
Supplement: S6 Table — Note: Functional annotation clustering analysis was performed using STRING. (DOCX) [file pone.0167212.s008.docx]

**Table S6. Functional annotation clustering analysis for the ‘overlapped’, ‘European-specific’, and ‘Asian-specific’ RA-associated genes**

|  | **#pathway ID** | **pathway description** | **observed gene count** | **false discovery rate** | **matching proteins(labels)** |
| --- | --- | --- | --- | --- | --- |
| **71 overlapped genes** | 4612 | Antigen processing and presentation | 4 | 6.32E-03 | HLA-A,HLA-DMA,HLA-G,HSP90AB1 |
|  | 5330 | Allograft rejection | 3 | 9.41E-03 | HLA-A,HLA-DMA,HLA-G |
|  | 5332 | Graft-versus-host disease | 3 | 9.41E-03 | HLA-A,HLA-DMA,HLA-G |
|  | 4940 | Type I diabetes mellitus | 3 | 9.70E-03 | HLA-A,HLA-DMA,HLA-G |
|  | 5320 | Autoimmune thyroid disease | 3 | 1.42E-02 | HLA-A,HLA-DMA,HLA-G |
|  | 5416 | Viral myocarditis | 3 | 1.67E-02 | HLA-A,HLA-DMA,HLA-G |
|  | 4145 | Phagosome | 4 | 1.77E-02 | HLA-A,HLA-DMA,HLA-G,TUBB |
| **76 European-specific genes** | 4740 | Olfactory transduction | 8 | 5.11E-03 | OR12D3,OR14J1,OR2B3,OR2H2,OR2J2,OR2J3,OR2W1,OR5V1 |
| **74 Asian-specific genes** | 5322 | Systemic lupus erythematosus | 18 | 1.79E-25 | HIST1H2AA,HIST1H2AC,HIST1H2AE,HIST1H2AH,HIST1H2AJ,HIST1H2AM,HIST1H2BA,HIST1H2BB,HIST1H2BD,HIST1H2BH,HIST1H2BI,HIST1H2BK,  HIST1H2BL,HIST1H2BM,HIST1H2BN,HIST1H2BO,HIST1H3F,HIST1H4G |
|  | 5034 | Alcoholism | 18 | 1.27E-22 | HIST1H2AA,HIST1H2AC,HIST1H2AE,HIST1H2AH,HIST1H2AJ,HIST1H2AM,HIST1H2BA,HIST1H2BB,HIST1H2BD,HIST1H2BH,HIST1H2BI,HIST1H2BK,  HIST1H2BL,HIST1H2BM,HIST1H2BN,HIST1H2BO,HIST1H3F,HIST1H4G |
|  | 5203 | Viral carcinogenesis | 11 | 1.08E-09 | HIST1H2BA,HIST1H2BB,HIST1H2BD,HIST1H2BH,HIST1H2BI,HIST1H2BK,  HIST1H2BL,HIST1H2BM,HIST1H2BN,HIST1H2BO,HIST1H4G |

Note: Functional annotation clustering analysis was performed using STRING.
